# Climate change impacts on rainfall intensity–duration–frequency curves in local scale catchments

**DOI:** 10.1007/s10661-024-12532-2

**Published:** 2024-03-15

**Authors:** Muyuan Xu, Lelys Bravo de Guenni, José Rafael Córdova

**Affiliations:** 1 University of Illinois at Urbana-Champaign, Champaign, IL USA; 2 Universidad Simón Bolívar, Caracas, Venezuela

**Keywords:** IDF curves, Climate models, Bootstrap, Clausius-Clapeyron relationship, SSP, Climate change, Rainfall extremes

## Abstract

The increasing intensity and frequency of rainfall events, a critical aspect of climate change, pose significant challenges in the construction of intensity–duration–frequency (IDF) curves for climate projection. These curves are crucial for infrastructure development, but the non-stationarity of extreme rainfall raises concerns about their adequacy under future climate conditions. This research addresses these challenges by investigating the reasons behind the IPCC climate report’s evidence about the validity that rainfall follows the Clausius-Clapeyron (CC) relationship, which suggests a 7% increase in precipitation per 1 °C increase in temperature. Our study provides guidelines for adjusting IDF curves in the future, considering both current and future climates. We calculate extreme precipitation changes and scaling factors for small urban catchments in Barranquilla, Colombia, a tropical region, using the bootstrapping method. This reveals the occurrence of a sub-CC relationship, suggesting that the generalized 7% figure may not be universally applicable. In contrast, our comparative analysis with Illinois, USA, an inland city in the north temperate zone, shows adherence to the CC relationship. This emphasizes the need for local parameter calculations rather than relying solely on the generalized 7% figure.

**Supplementary Information:**

The online version contains supplementary material available at 10.1007/s10661-024-12532-2.

## Introduction

Graphical tools known as historical intensity–duration–frequency (IDF) curves are commonly employed to depict the probability of various extreme rainfall events. These curves are commonly used and developed by various agencies around the world using statistical methods and historical rainfall data. In stormwater infrastructure design, IDF curves are widely utilized to capture the properties of extreme rainfall events (Cook et al., 2020). However, there is a growing recognition among numerous organizations about the necessity to update IDF curves to account for anticipated alterations in rainfall patterns caused by climate change (Bibi & Tekesa, 2023; Kourtis et al., 2022; Martel et al., 2021; Şen & Kahya, 2021).

There are several adaptation strategies that provide standards for the modification of IDF curves to account for the future climate. One simplest strategy is to apply a constant percentage increase on all rainfall extremes (Madsen et al., 2014). Another strategy is to apply adaptive percentage increase based on various factors, such as local temperature and frequency of the rainfall (Gregersen et al., 2014). The third one is to apply percentage increase based on the projected increase in temperature, while a scaling parameter in the equation follows the rationale of Clausius-Clapeyron relationship.

The future intensity of precipitation can be estimated using the following equation (Martel et al., 2021):

1 $${I}_{fut}={I}_{ref}\times {\left[\frac{100+{R}_{sc}}{100}\right]}^{\Delta T}$$

In this equation, ${I}_{fut}$ represents the future rainfall intensity, ${I}_{ref}$ denotes the reference rainfall intensity, $\Delta T$ represents the projected local temperature change, and ${R}_{sc}$ represents the rainfall scaling factor based on the Clausius-Clapeyron relationship. The Canadian Standard Association (CSA, 2019) recommends a 7% increase in rainfall intensity per degree Celsius increase in temperature, aligning with the conclusion from the IPCC report that states, “the water-holding capacity of the atmosphere increases by about 7% for every 1 °C rise in temperature” (IPCC, 2007). The second part of the right-hand side in the Eq. (1) is regarded as the correction factor.

Many observational and modeling studies have explored the link between annual maximum daily precipitation and atmospheric temperature, consistently finding a scaling rate approximating the Clausius-Clapeyron rate of around 7% per degree Celsius, with a possible lower rate in drier land regions (O’Gorman & Muller, 2010; Sherwood et al., 2010; Simmons et al., 2010; Westra et al., 2013; Willett et al., 2007). Despite the widespread acceptance of the 7% increase in atmospheric water content per 1 °C temperature rise, its validity is not without complexity. Moreover, since we will be comparing this conclusion with our own calculation, it is necessary to critically assess and deduce conclusions from this commonly accepted assumption. The widely recognized Clausius-Clapeyron relation pertains to the gradient of any phase boundary line within a Pressure–Temperature diagram, encompassing the boundary that distinguishes between the liquid and gas phases. In our context, this relation explains the equilibrium between water vapor and liquid water, consequently illustrating the atmosphere’s ability to retain moisture.

The derivation of the Clausius-Clapeyron relationship and the derivation of the 7% coefficient in Physics are elaborated upon in Appendix A.

The objective of this research is to assess the impact of climate change on the IDF curves of extreme rainfall events lasting less than 24 h in small urban catchments within a tropical region. The specific study area is Barranquilla, Colombia, situated at coordinates 11.04° North latitude and − 74.82° West longitude. The adopted methodology is adapted from the approach proposed by Martel et al. (2021). Martel’s study, while comprehensive, is based on a global or coarse scale. In contrast, our research focuses on local conditions, providing a more detailed and nuanced understanding of precipitation patterns. Furthermore, our study places a greater emphasis on the analysis of IDF curves. This allows us to provide more precise guidelines for infrastructure development and climate adaptation strategies. Historical simulations for the IDF curves are available for this location (known as the Reference IDF). A correction factor, accounting for future temperature and precipitation increases under four different Shared Socioeconomic Pathways (SSPs), will be developed for this location. This correction factor will be compared to the parameter derived from the Clausius-Clapeyron relationship, where ${R}_{sc}$ is approximately 7%.

We present the source of dataset and data processing methods in the “Dataset and data processing” section, followed by a demonstration of our results in the “Results” section. The “Discussion” section provides discussions and insights derived from our findings, while the “Conclusion” section offers a comprehensive conclusion and implications.

## Dataset and data processing

Global climate models are state-of-the-art tools to examine future projections of climate variables under different scenarios of future economic development and greenhouse gas emissions. Other approaches as machine learning methods including long-short term memory (LSTM) and random forest have been proposed for monthly rainfall forecasting (Chen et al., 2022) which could handle any global climatic conditions. Urban flood risk management using high resolution digital sensors (Jiang et al., 2023) or deterministic model-based simulations have been proposed by some authors to handle stormwater design complexities at the local scale (Tansar et al., 2023).

For our study, we rely on data obtained from global climate model (GCM) projections. Specifically, we use the CMIP6 data, which is currently not globally downscaled and bias-corrected by the World Climate Research Program (WCRP). To access the downscaled data, we download it from the GISS Model Output Data Server, which is affiliated to the NASA NCCS data server. The specific dataset we utilize is the NASA Earth Exchange (NEX) Global Daily Downscaled Projections (GDDP) dataset. The NEX-GDDP-CMIP6 dataset is generated using the bias-correction spatial disaggregation (BCSD) method, which is a statistical downscaling algorithm designed specifically to overcome the limitations associated with GCM outputs (NASA, 2022). Importantly, each climate projection within this dataset has been downscaled to a spatial resolution of 0.25° × 0.25°.

In our analysis, we focus on the mean daily temperature and daily precipitation records from the dataset. We extract data from 35 climate models in the dataset and extract only the record whose coordinates are located at the coordinates 11.04°N, − 74.82°W, which correspond to the study location of Barranquilla, Colombia. The time scale of the data covers two distinct periods: 1981–2010, representing the historical period, and 2071–2100, representing the projection period. We ensure that the lengths of these time periods are consistent to facilitate comparisons between historical and projected data.

We specifically consider the far future projection period for our analysis for two main reasons. Firstly, examining the far future allows us to anticipate more extreme changes in precipitation patterns, providing a more dramatic contrast to historical data and theoretical predictions, and thus enables us to better understand the potential impacts of climate change on rainfall events. Secondly, infrastructure developments are long-term investments, often designed to last for several decades. Therefore, considering only near or mid-future periods may not provide an adequate basis for infrastructure.

The complete dataset comprises data from 35 CMIP6 GCMs, incorporating both historical experiments and four SSP scenarios (SSP1-2.6, SSP2-4.5, SSP3-7.0, and SSP5-8.5). However, it is worth noting that one model, TaiESM1, deviates significantly in temperatures from all other models across all SSPs. Considering these discrepancies, the results obtained from TaiESM1 are considered outliers and are treated accordingly in our analysis.

It is important to note that not all models within the dataset have data available for all four SSP scenarios. Specifically, we have data from 35 models for SSP5-8.5, 28 models for SSP3-7.0, 34 models for SSP2-4.5, and 32 models for SSP1-2.6.

Additionally, there are four models within the dataset that have data available for all 30 days of every month, resulting in a total of 360 days for each year. To ensure consistency and accuracy in the dataset, certain adjustments were made. First, records for February 30th were removed, as well as data for February 29th in non-leap years. For missing days such as January 31st, estimates were calculated by taking the average of the neighboring records. For example, the temperature for May 31, 2081, was calculated as the average of the temperatures on May 30, 2081, and June 1, 2081.

There are multiple models that do not have records for February 29th in leap years. Similarly, for these missing days, estimates were generated using the same method of averaging neighboring days.

It is worth mentioning that one model, IITM-ESM, has missing data for January 1st in multiple years, the entire year 2100, and the entire year 2099 in SSP3-7.0. To address this, missing data for January 1st were generated using the same averaging method applied to neighboring days. However, data for the year 2100 and the year 2099 in SSP3-7.0 is not considered for this model in our calculations.

To incorporate adaptive IDF for future climate change considerations, Eq. (1) from Martel et al. (2021) undergoes modifications. The adapted equation (Eq. 2) is as follows:

2 $${I}_{fut,D,T}={I}_{ref,D, T}\times {F}_{T}\times {F}_{D}\times {\left(\frac{100+{R}_{sc,\mathrm{24,2}}}{100}\right)}^{\Delta T}$$

where ${I}_{fut,D,T}$ represents the projected future rainfall intensity for a given duration D and return period T. ${I}_{ref,D,T}$ represents the reference period rainfall intensity for the same duration D and return period T. ${F}_{T}$ is the adjustment factor for the return period T, where ${F}_{T}$ is equal to or greater than 1 for return periods longer than 2 years ($T > 2$ years). ${F}_{D}$ is the adjustment factor for durations shorter than 24 h ($D < 24 hours$), where ${F}_{D}$ is equal to or greater than 1. ${R}_{sc,\mathrm{24,2}}$ represents the rainfall scaling factor (%/°C) for the 24-h, 2-year return period rainfall event. $\Delta T$ represents the projected change in seasonal mean temperature (in degrees Celsius).

${F}_{D}$ and ${F}_{T}$ are both positive factors, each having a value of 1 or greater. As the duration of rainfall diminishes (compared to the 24-h reference duration) or the return period of rainfall increases (beyond the 2-year reference return period), these factors progressively increase above 1. A value of 1 signifies that there is no magnification of rainfall extremes for durations shorter than 24 h (${F}_{D}$) or return periods longer than 2 years (${F}_{T}$). It is noteworthy that Martel et al. (2021) caution against a simple application of the ~ 7% increase per degree Celsius rate at the regional level. They highlight evidence of an increased Clausius-Clapeyron rate for shorter duration extremes (hourly or sub-daily) when temperatures fall within the range of approximately 12 to 22 °C. Conversely, there is also evidence of a limitation, or even a decrease, in precipitation intensity with increasing temperatures above approximately 24 °C, and Barranquilla experiences monthly temperature averages above 24 °C. However, it is important to acknowledge that obtaining reliable estimates of the ${F}_{D}$ and ${F}_{T}$ factors is challenging due to the unavailability of NEX-GDDP-CMIP6 data for durations less than 24 h. For this reason, in our study, we consider values equal to 1 for these two factors.

Furthermore, Eq. (2) relies on the rainfall scaling factor for the 24-h, 2-year return period event. This event was chosen because it represents the lowest intensity in the IDF curves. Calculating the rainfall scaling factor for this event is relatively straightforward since we only need the record of the median of maximum daily rainfall (Rx1day). To illustrate the median is of interest to us, consider the values equivalent to the maximum daily precipitation recorded within a single year. From 2071 to 2100, we have 30 numbers representing maximum daily rainfall (Rx1day). For the median among those numbers, there are half of the numbers that are greater than the median, so that the exceedance probability is equal to 50%, meaning that the return period for the median is 2 years. By comparing the medians of Rx1day between future and past periods, we can estimate the change in precipitation. Similarly, by comparing the mean temperatures between future and past periods, we can estimate the temperature change. This allows us to calculate ${R}_{sc}$ as the rate of precipitation change (expressed in percentage) divided by temperature change. Thus, we only need the median of Rx1day in precipitation and the mean of temperature to calculate the ${R}_{sc}$, which quantifies the rate of precipitation change in relation to temperature change.

## Results

After the data-cleaning process, the focus shifts to extracting the mean daily temperature and daily precipitation records from the NEX-GDDP-CMIP6 dataset at the designated coordinates (11.04°N, − 74.82°W). The updated dataset now includes four SSP scenarios, with each scenario comprising a certain number of climate models.

To proceed with the analysis, the next step is to extract the daily precipitation and temperature data for each climate model under a specific SSP scenario. The data extraction covers two distinct time periods: 1981 to 2010 for historical data and 2071 to 2100 for projected data. It is important to note that the historical dataset is derived from CMIP6 simulations rather than actual historical data. This approach is chosen because our insights into future climate patterns are gained from computational climate models. Although forecasting precise day-to-day weather conditions over a 50-year horizon remains nearly impossible, we can, nonetheless, assess whether forthcoming decades are expected to exhibit heightened temperatures relative to our contemporary climate, as proposed by Miller et al. (2021). Within each dataset, we further refine the analysis by computing the maximum daily precipitation and the mean daily temperature for each year. As a result, for each climate model under a specific SSP scenario, we have a dataset with 60 rows and 2 columns. Each row represents a year, while the two columns capture the maximum precipitation recorded during that year and the mean temperature observed throughout the year.

### Precipitation

To visualize the precipitation data for each climate model, we generate five spaghetti plots. These plots depict the variability in precipitation over time for different time periods. It is important to note that the historical data includes records from all 35 climate models, SSP5-8.5 includes 35 models, SSP3-7.0 includes 28 models, SSP2-4.5 includes 34 models, and SSP1-2.6 includes 32 models.

**Fig. 1:**
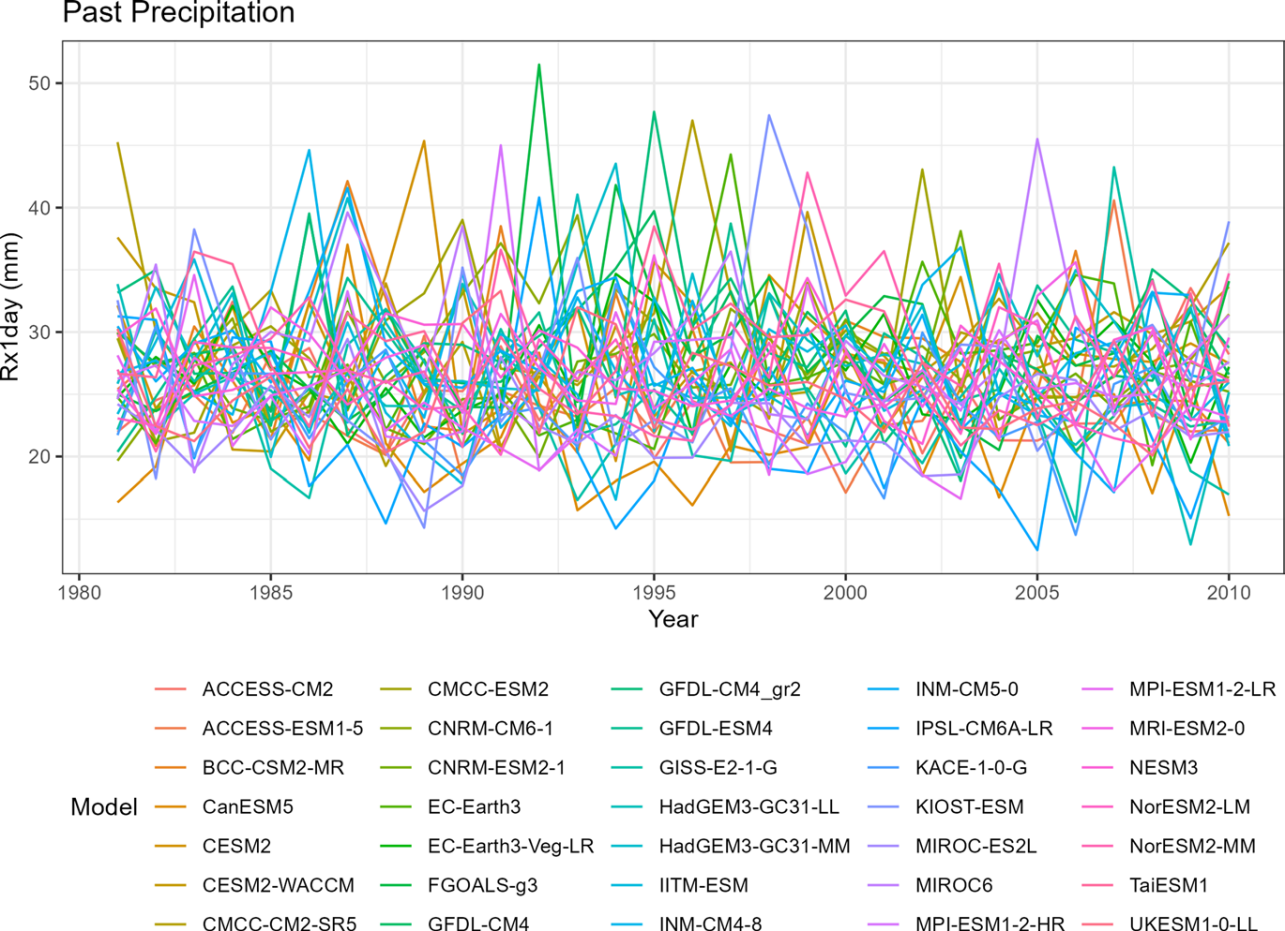
Annual maximum daily precipitation (Rx1day) of historical simulations

**Fig. 2:**
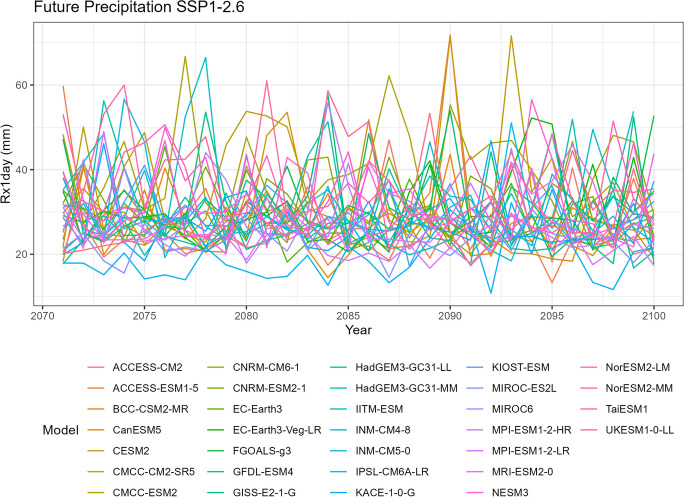
Annual maximum daily precipitation (Rx1day) of projected data SSP1-2.6

**Fig. 3:**
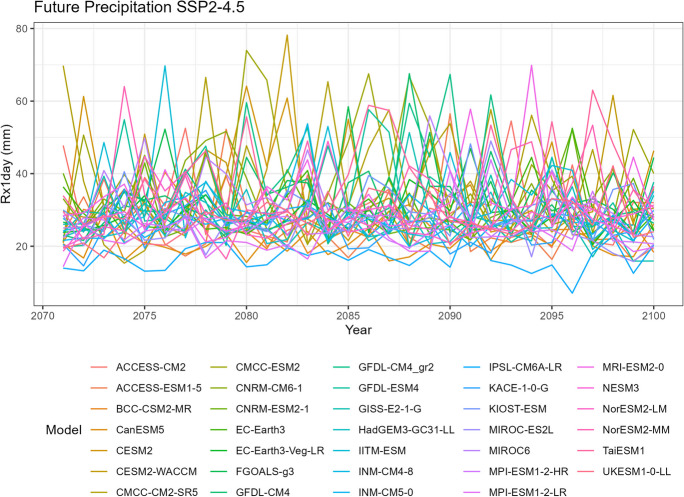
Annual maximum daily precipitation (Rx1day) of projected data SSP2-4.5

**Fig. 4:**
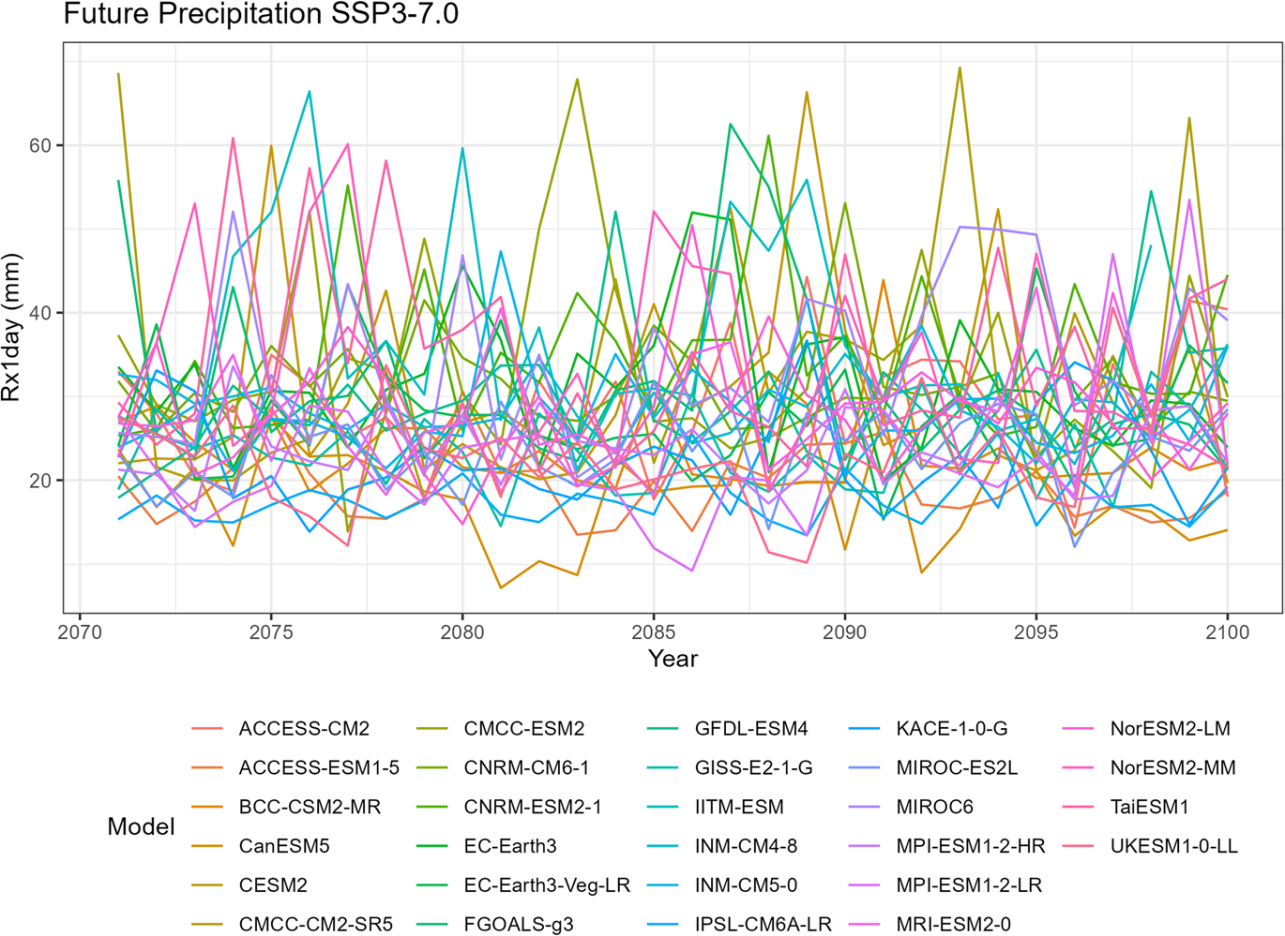
Annual maximum daily precipitation (Rx1day) of projected data SSP3-7.0

**Fig. 5:**
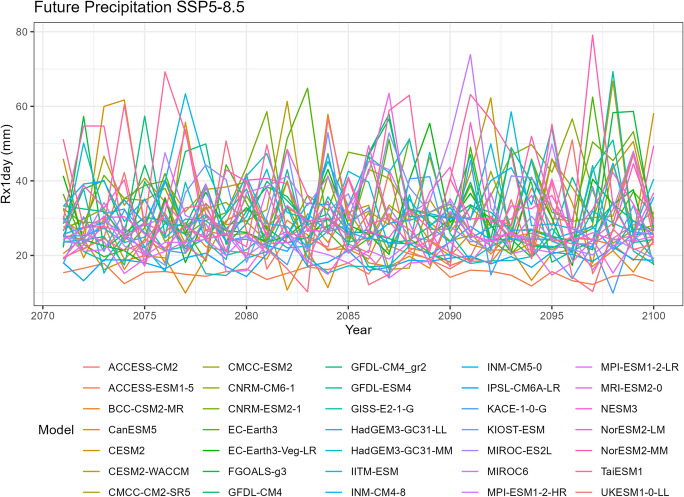
Annual maximum daily precipitation (Rx1day) of projected data SSP5-8.5

The first spaghetti plot (Fig. 1) is based on historical simulations, representing the annual maximum daily (Rx1day) precipitation patterns simulated during the period from 1981 to 2010. The remaining four spaghetti plots (Figs. 2, 3, 4, and 5) are based on the projected data under the four different SSP scenarios. These plots illustrate the projected annual maximum daily precipitation patterns from 2071 to 2100 for each SSP scenario. Each spaghetti plot consists of multiple lines, with each line representing the annual Rx1day precipitation values for a specific climate model. By comparing these plots, we can observe the inter-model variability and assess the potential changes in precipitation patterns under different SSP scenarios.

### Temperature

**Fig. 6:**
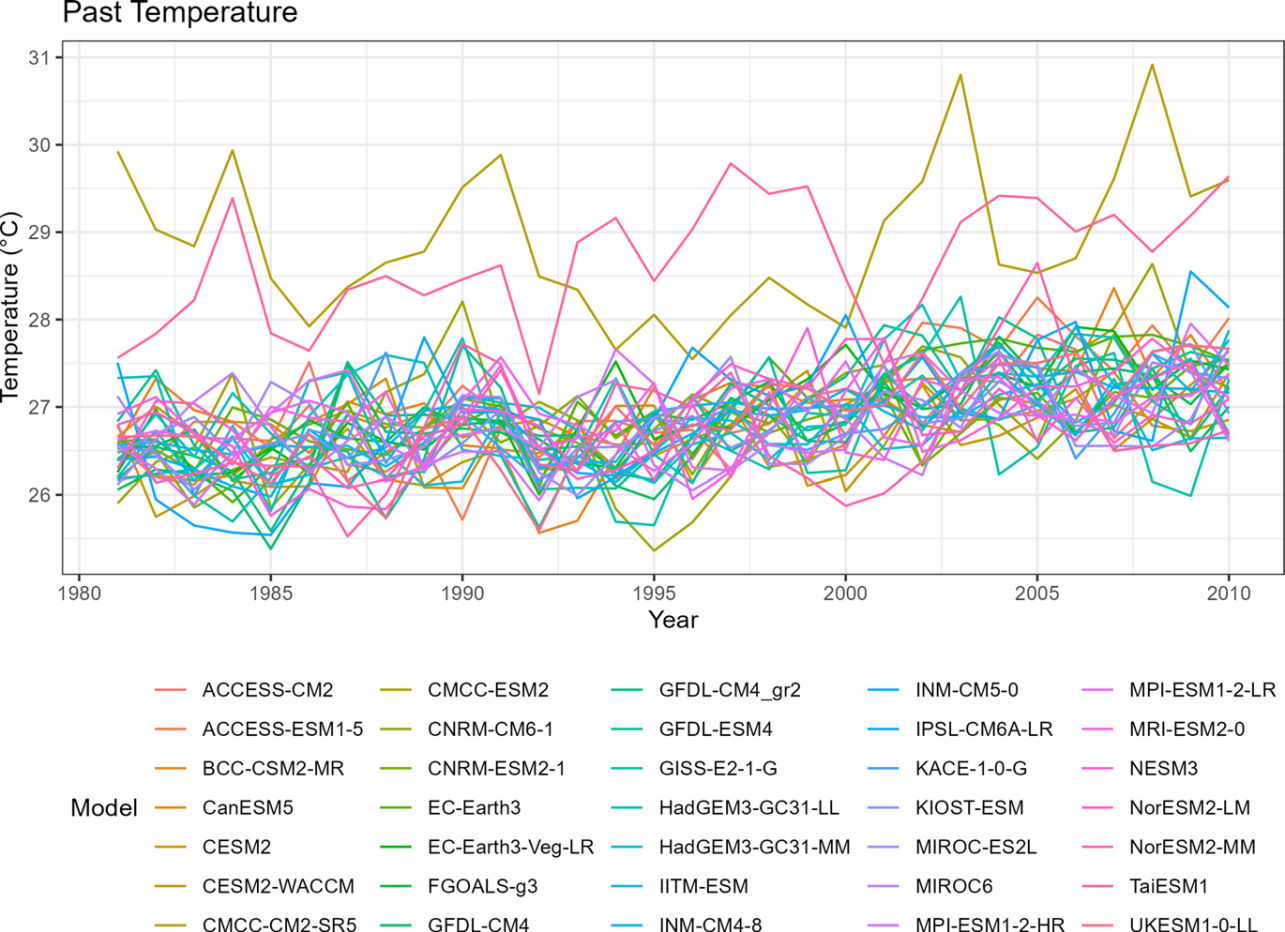
Annual mean temperature of historical simulations

**Fig. 7:**
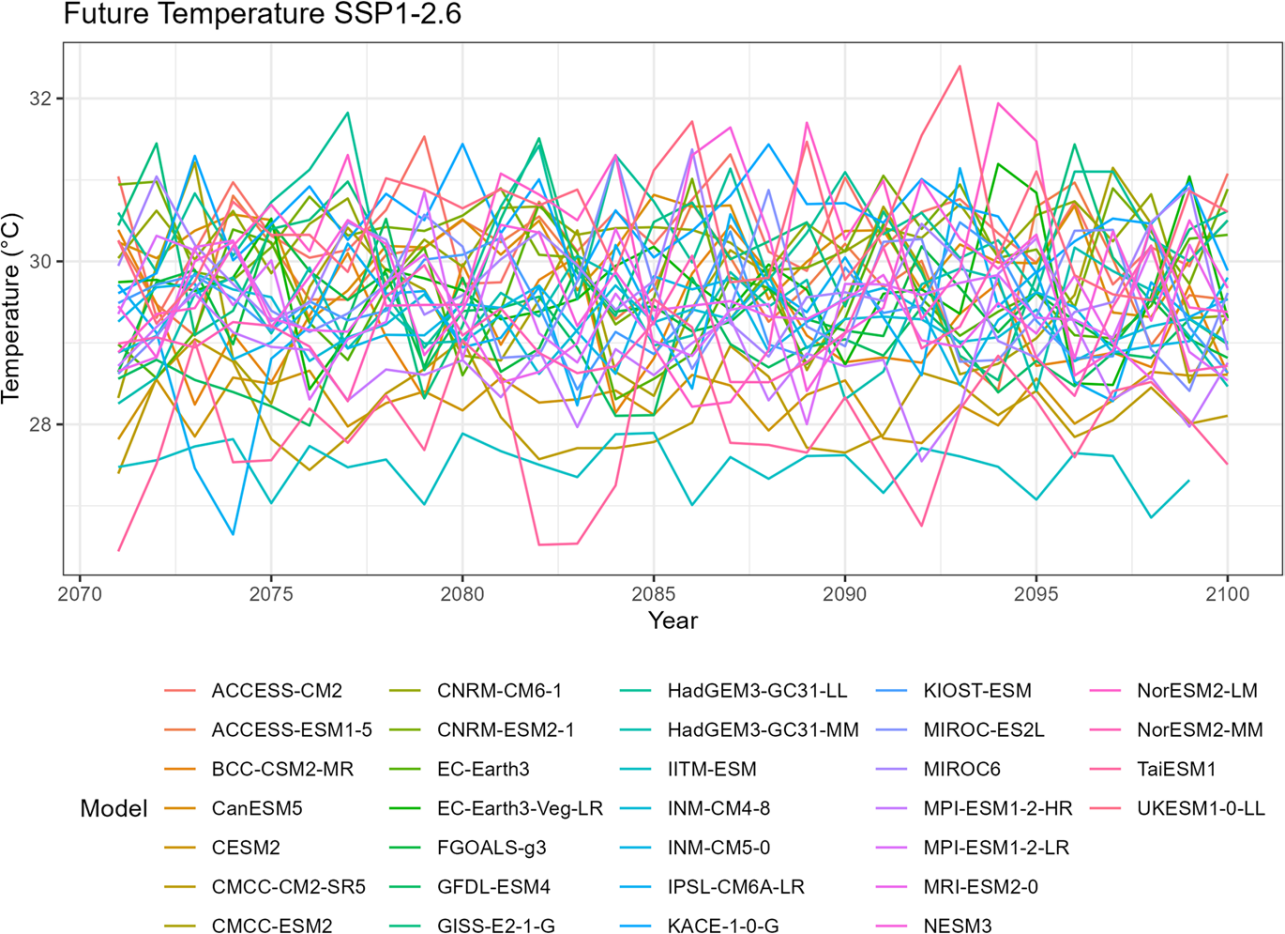
Annual mean temperature of projected data SSP1-2.6

**Fig. 8:**
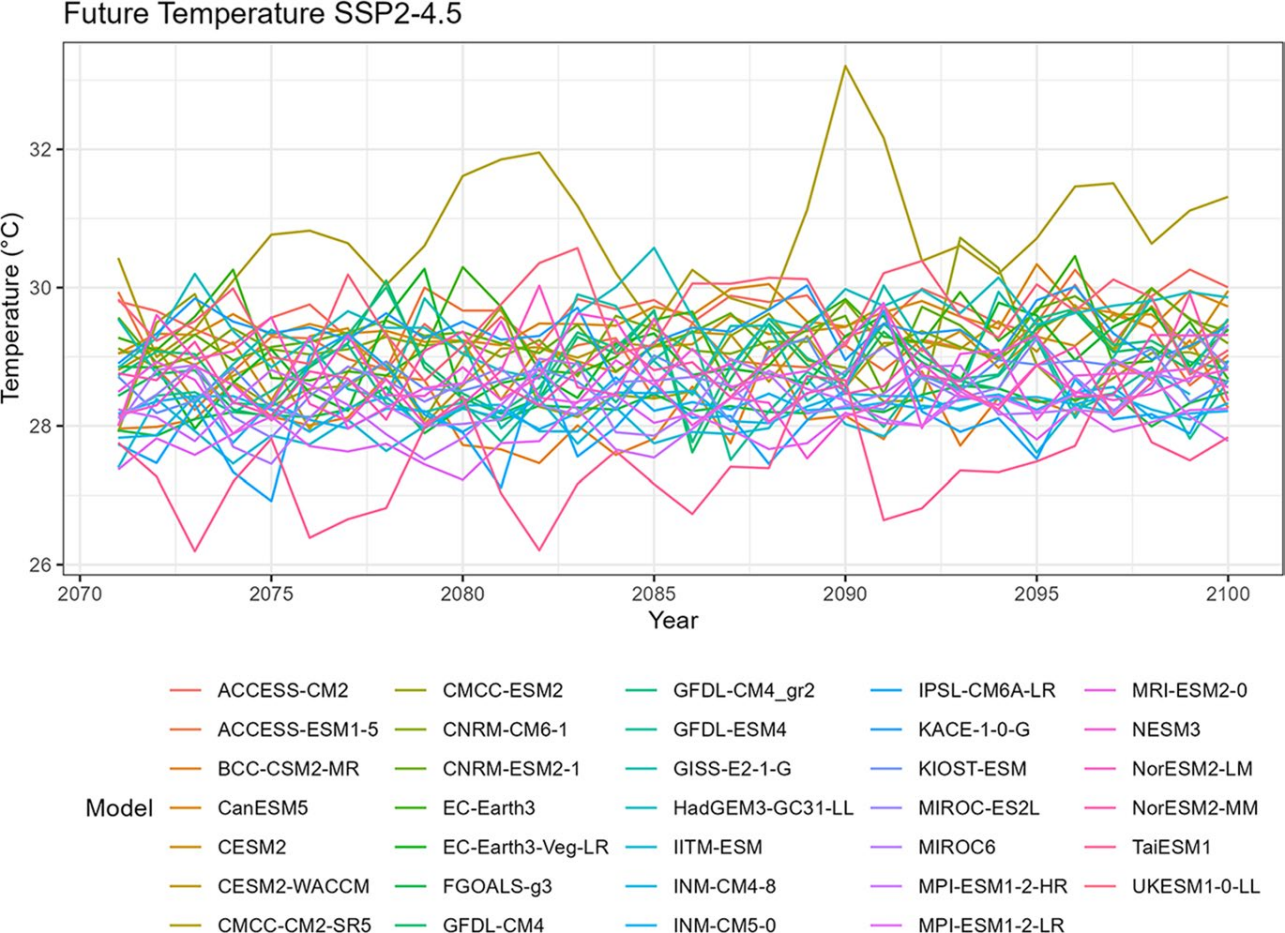
Annual mean temperature of projected data SSP2-4.5

**Fig. 9:**
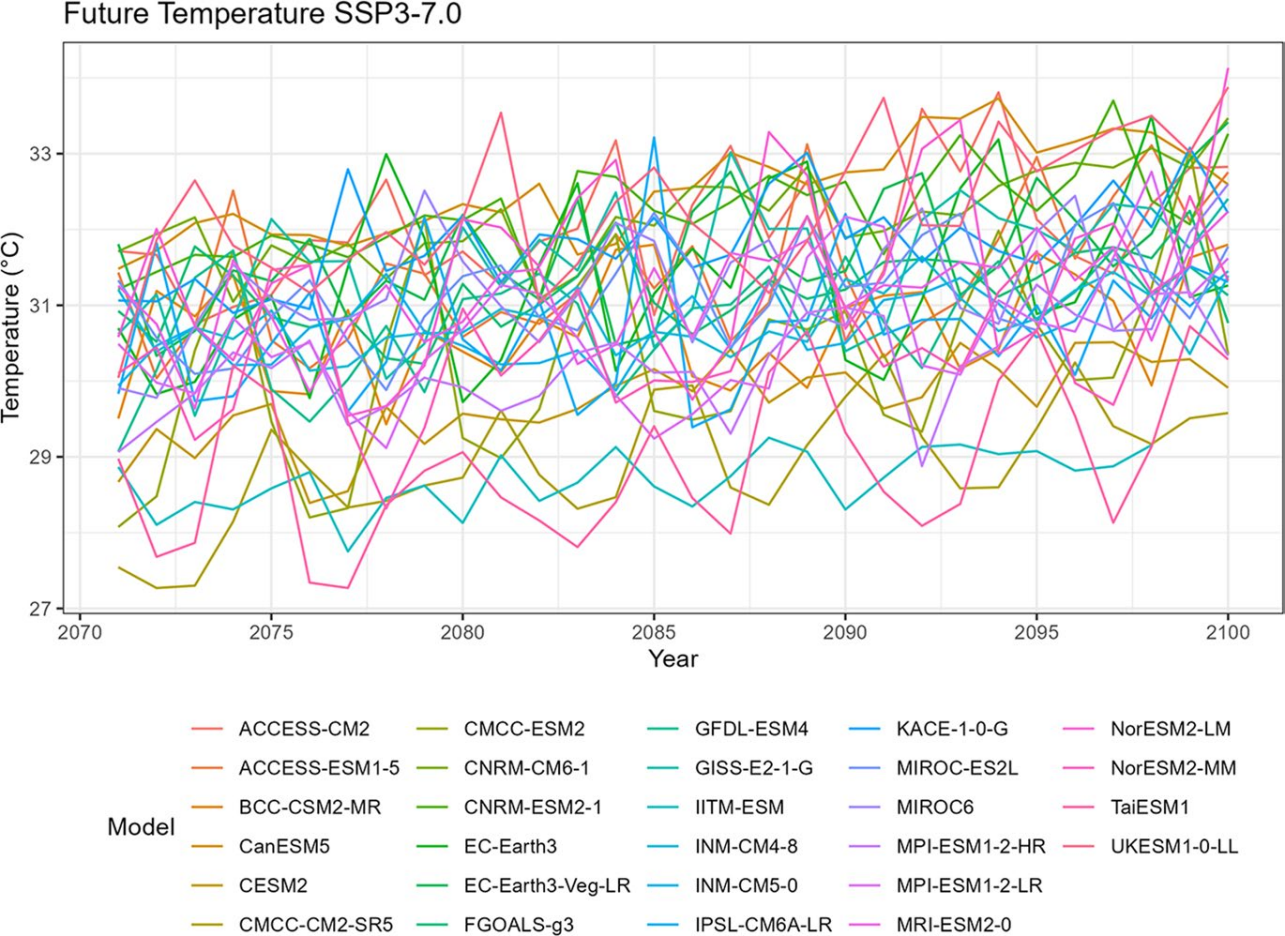
Annual mean temperature of projected data SSP3-7.0

**Fig. 10:**
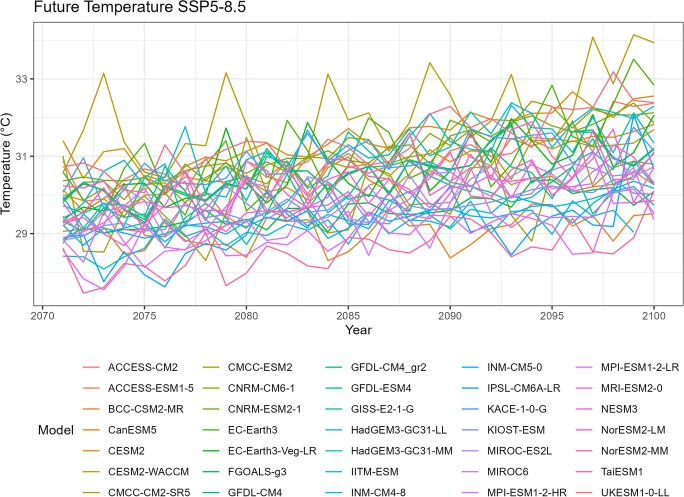
Annual mean temperature of projected data SSP5-8.5

Similar to the precipitation data, spaghetti plots can also be generated for annual mean temperature to visualize the variability over time (Figs. 6, 7, 8, 9, and 10). It is worth noting that the plots also highlight any outliers or deviations, such as the issue observed with the TaiESM1 model mentioned in the tech note.

### ${R}_{sc}$ and the correction factor

As explained in the “Dataset and data processing” section, ${R}_{sc}$ represents the rate of precipitation change relative to temperature change. Equation (2) is utilized in this research to calculate the ${R}_{sc}$ value specifically for the 24-h, 2-year return period rainfall event (${R}_{sc,\mathrm{24,2}}$), and to calculate the correction factor $CF={\left(\frac{100+{R}_{sc,\mathrm{24,2}}}{100}\right)}^{\Delta T}$.

To calculate ${R}_{sc,\mathrm{24,2}}$ for each climate model within a given SSP scenario using the dataset consisting of 60 rows and 2 columns, the following steps are involved:

1. Extract the median value of Rx1day for the past period (1981–2010) as $P{r}_{1}$.
2. Extract the median value of Rx1day for the future period (2071–2100) as $P{r}_{2}$.
3. Determine the mean temperature for the past period (1981–2010) as ${T}_{1}$.
4. Determine the mean temperature for the future period (2071–2100) as ${T}_{2}$.

Then, we can have our value of ${R}_{sc,\mathrm{24,2}}$ along with the corresponding correction factor CF:

$${R}_{sc,\mathrm{24,2}}=\frac{\frac{P{r}_{2}-P{r}_{1}}{P{r}_{1}}\times 100\%}{\Delta {\text{T}}}=\frac{(P{r}_{2}-P{r}_{1})/P{r}_{1}\times 100\%}{{T}_{2}-{T}_{1}}$$

**Fig. 11:**
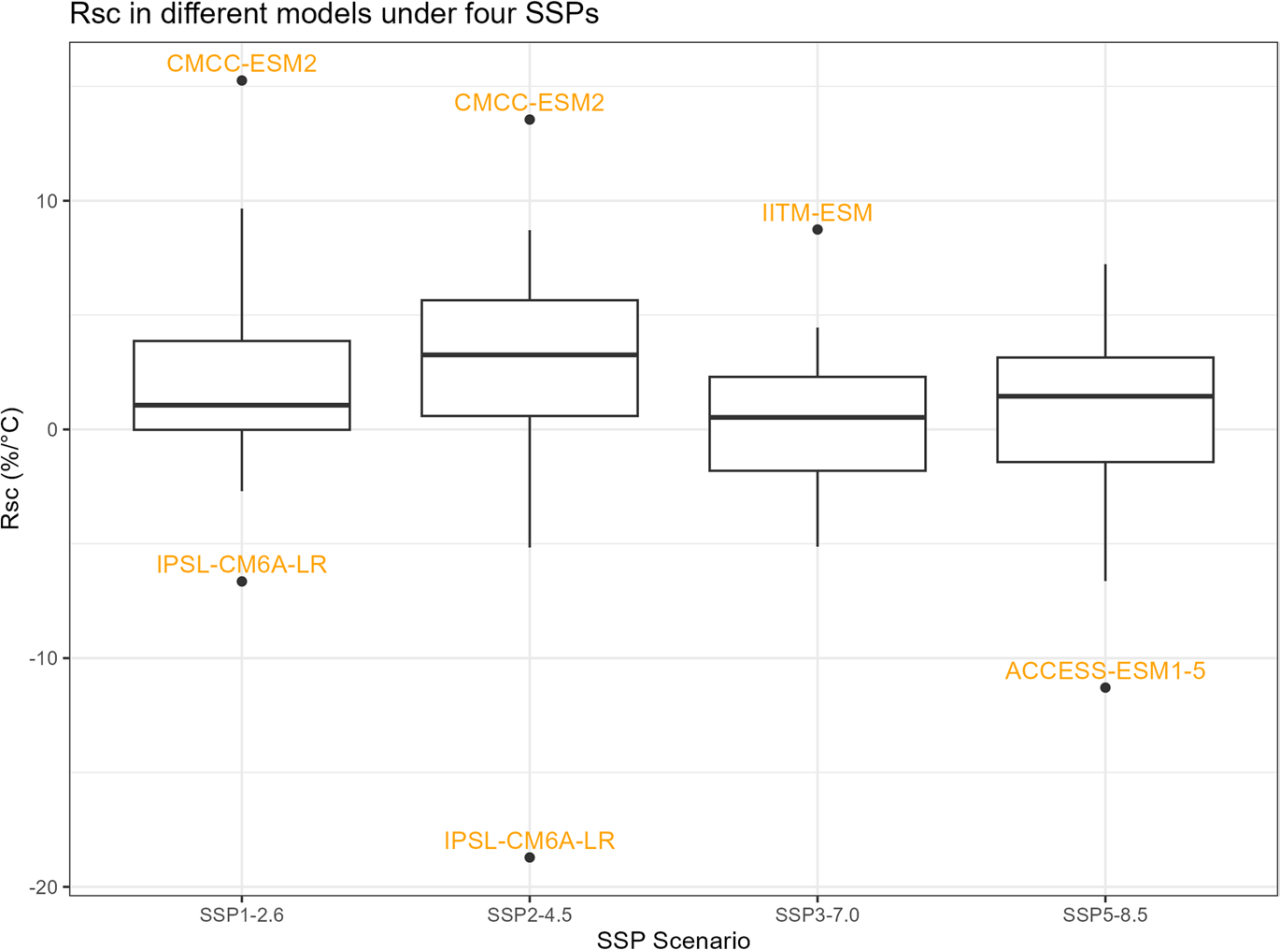
${R}_{sc}$ of each model under four SSPs

**Fig. 12:**
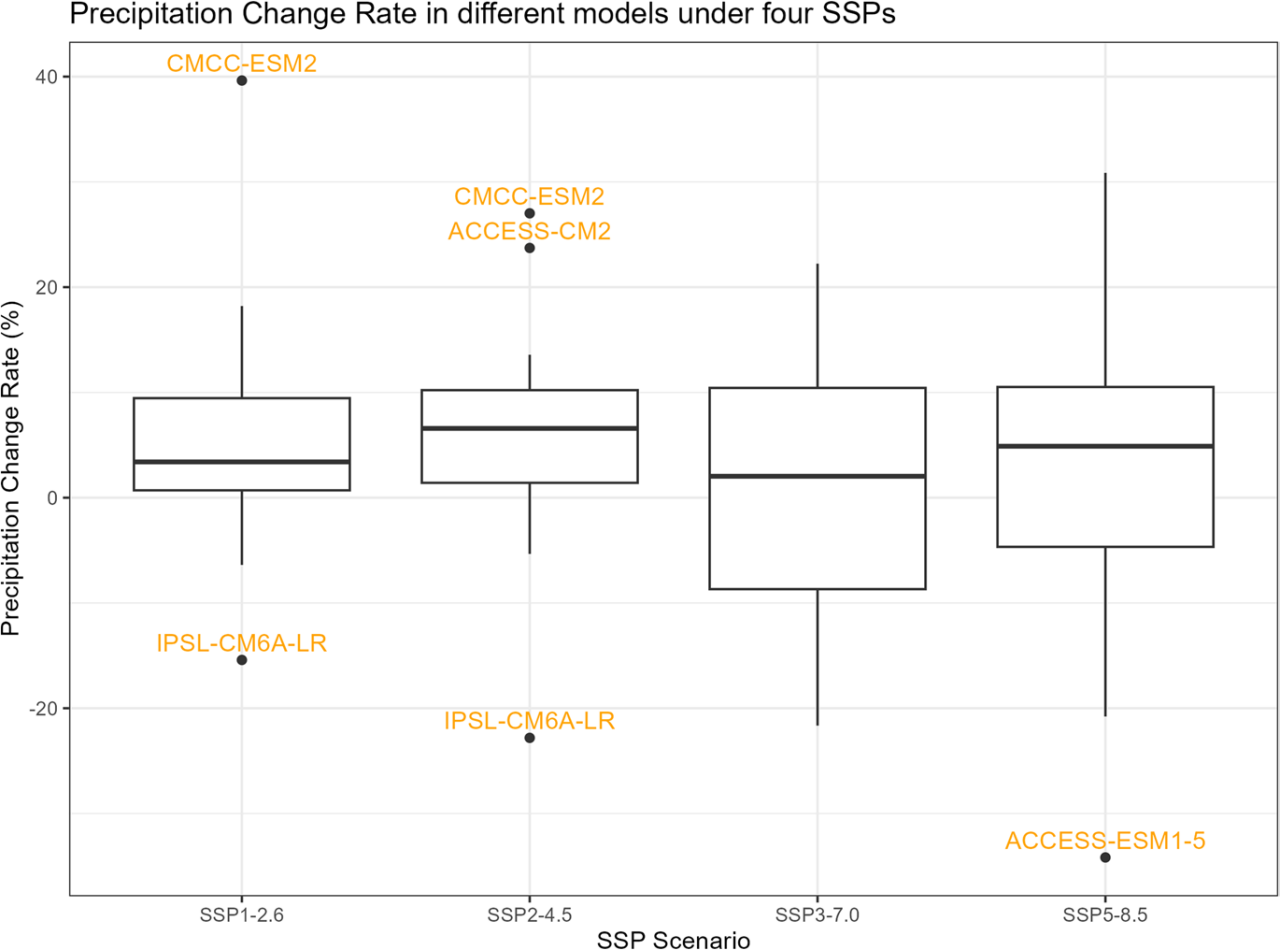
Precipitation change rate of each model under four SSPs

**Fig. 13:**
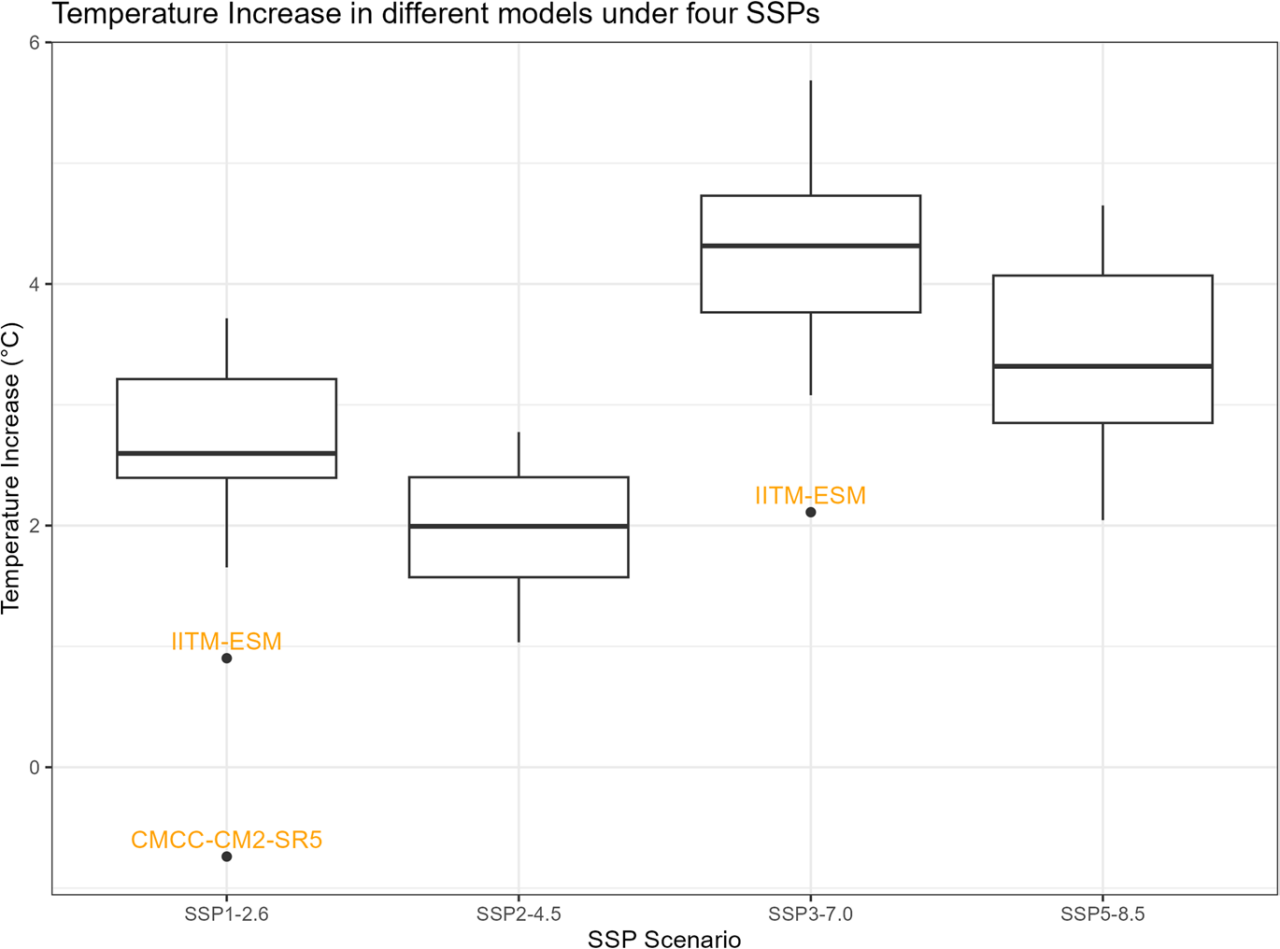
Temperature increase of each model under four SSPs

**Fig. 14:**
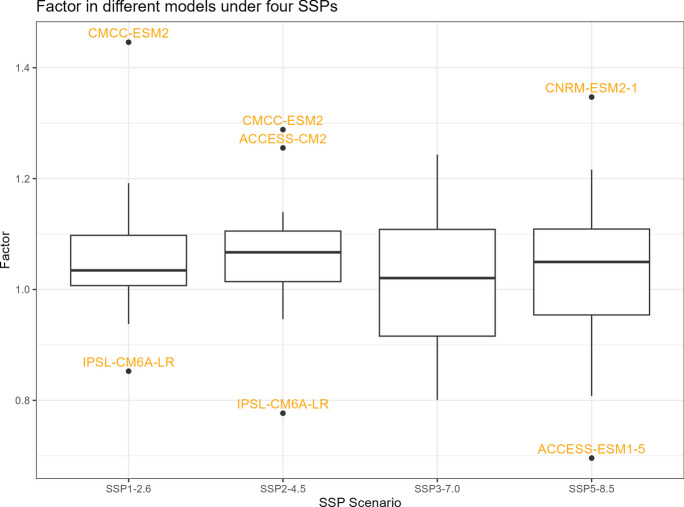
Correction factor of each model under four SSPs

The boxplots of ${R}_{sc,\mathrm{24,2}}$, percentage changes in precipitation, temperature change, and correction factor under four SSPs are shown in Figs. 11, 12, 13, and 14. However, it is important to note that the following histograms exclude Model TaiESM1 because of the model’s significant deviation in temperature from all other models across all SSP scenarios. Also, under SSP3-7.0, Model CMCC-CM2-SR5 has shown significant deviation from all other models, so it is also excluded from the result of this SSP scenario. To summarize the results, Tables 1 and 2 below provide summary tables of the ${R}_{sc,\mathrm{24,2}}$ and temperature increase, respectively, for each SSP scenario, while Tables 3 and 4 in Appendix B provide summary tables of the precipitation change rate and correction factor values.

**Table 1:** Summary of ${R}_{sc}$

SSPS	Min	Median	Mean	Max
SSP1-2.6	− 6.65	1.06	2.14	15.26
SSP2-4.5	− 18.72	3.26	2.51	13.55
SSP3-7.0	− 5.13	0.53	0.35	8.74
SSP5-8.5	− 11.29	1.45	0.40	7.23

**Table 2:** Summary of temperature change

SSPS	Min	Median	Mean	Max
SSP1-2.6	− 0.74	2.60	2.60	3.72
SSP2-4.5	1.03	1.99	1.98	2.77
SSP3-7.0	5.68	4.32	4.26	5.68
SSP5-8.5	2.04	3.32	3.39	4.65

Table 1 displays that the median ${R}_{sc}$ value for SSP2-4.5 is more than twice that of other SSPs. To gain deeper insights, we can analyze the components of the ${R}_{sc}$ equation, examining the numerator (precipitation change rate) and denominator (temperature increase). Looking at Fig. 12, which presents the boxplot of precipitation change, it is evident that there are no substantial differences in the mean change rate of precipitation among the various SSPs, except for SSP3-7.0, which exhibits greater variation. Figure 13, portraying the boxplot of temperature increase, shows distinctions among the SSPs, indicating the potential source of the differences in ${R}_{sc}$ values. Surprisingly, SSP2-4.5 has the least temperature increase, resulting in the highest ${R}_{sc}$ value. This observation seemingly contradicts intuition, as SSP1-2.6, characterized by highest sustainability, is expected to have least temperature increase because higher sustainability leads to less warming in climate change.

An intriguing observation arises from the results presented in Table 2. Contrary to our initial expectations and the findings in the Intergovernmental Panel on Climate Change (IPCC) Working Group I (WGI) Interactive Atlas, we observe that SSP3-7.0 > SSP5-8.5 > SSP1-2.6 > SSP2-4.5 in terms of the median and mean of temperature change among the four SSPs. This discrepancy could potentially be attributed to differences in spatial resolution. The IPCC Atlas mentions that “The CMIP5, CMIP6, and CORDEX datasets have been re-gridded to common 2°, 1°, or 0.5° resolutions.” In contrast, the dataset we employ in our study has a much higher spatial resolution (0.25° × 0.25°). Consequently, suppose the original dataset for our study area has a resolution of 2°, the increased spatial resolution in our downscaled dataset (64 times higher) could reasonably account for the variance in our results compared to the IPCC Atlas. Notably, the Atlas categorizes Barranquilla as a “low model agreement” area, which signifies that fewer than 80% of models agree on the direction of change, indicating substantial uncertainty. The downscaling method used to produce the NEX-GDDP from the CIMIP6 data set, which involves bias correction/spatial disaggregation and bi-linear interpolation (Thrasher et al., 2022), could contribute to the discrepancy. The other factor contributing to the discrepancy in the results, besides downscaling and postprocessing of climate simulations, could be the internal climate variability (unforced variability due to the chaotic nature of the climate system) (Martel et al., 2021), which generates the epistemic uncertainty of the model structure in future extreme rainfall changes.

To establish confidence intervals for ${R}_{sc}$ and its associated correction factor, a robust approach is imperative due to the limited number of records available in our dataset for each model under a specific SSP. Given that only 30 records exist in each time period for one model under one SSP, a resampling strategy becomes essential. This entails resampling the data multiple times and performing inference about a sample from the resampled data.

**Fig. 15:**
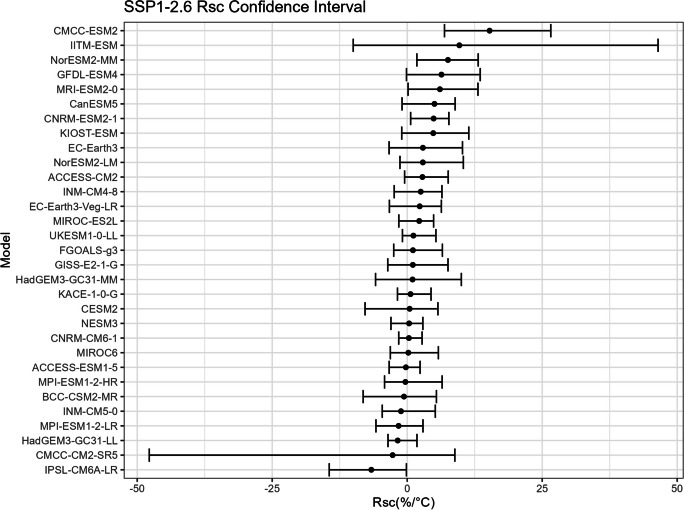
Confidence interval for ${R}_{sc}$ under SSP1-2.6

**Fig. 16:**
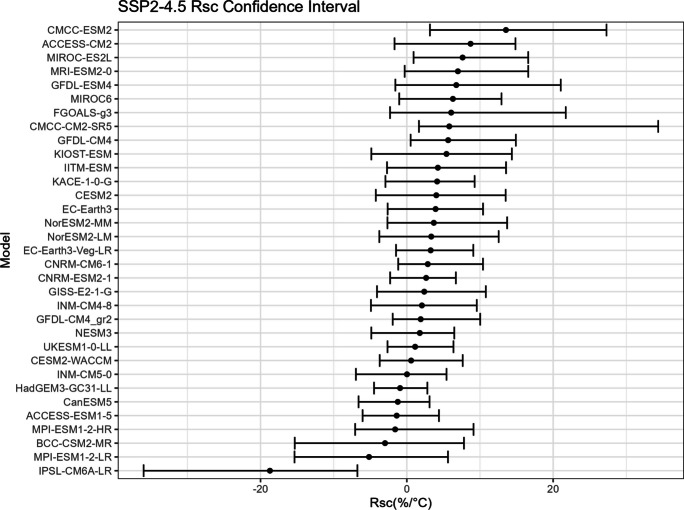
Confidence interval for ${R}_{sc}$ under SSP2-4.5

**Fig. 17:**
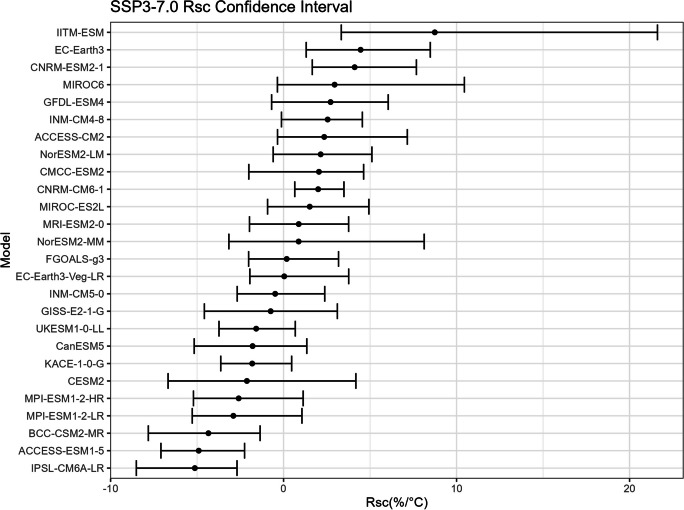
Confidence interval for ${R}_{sc}$ under SSP3-7.0

**Fig. 18:**
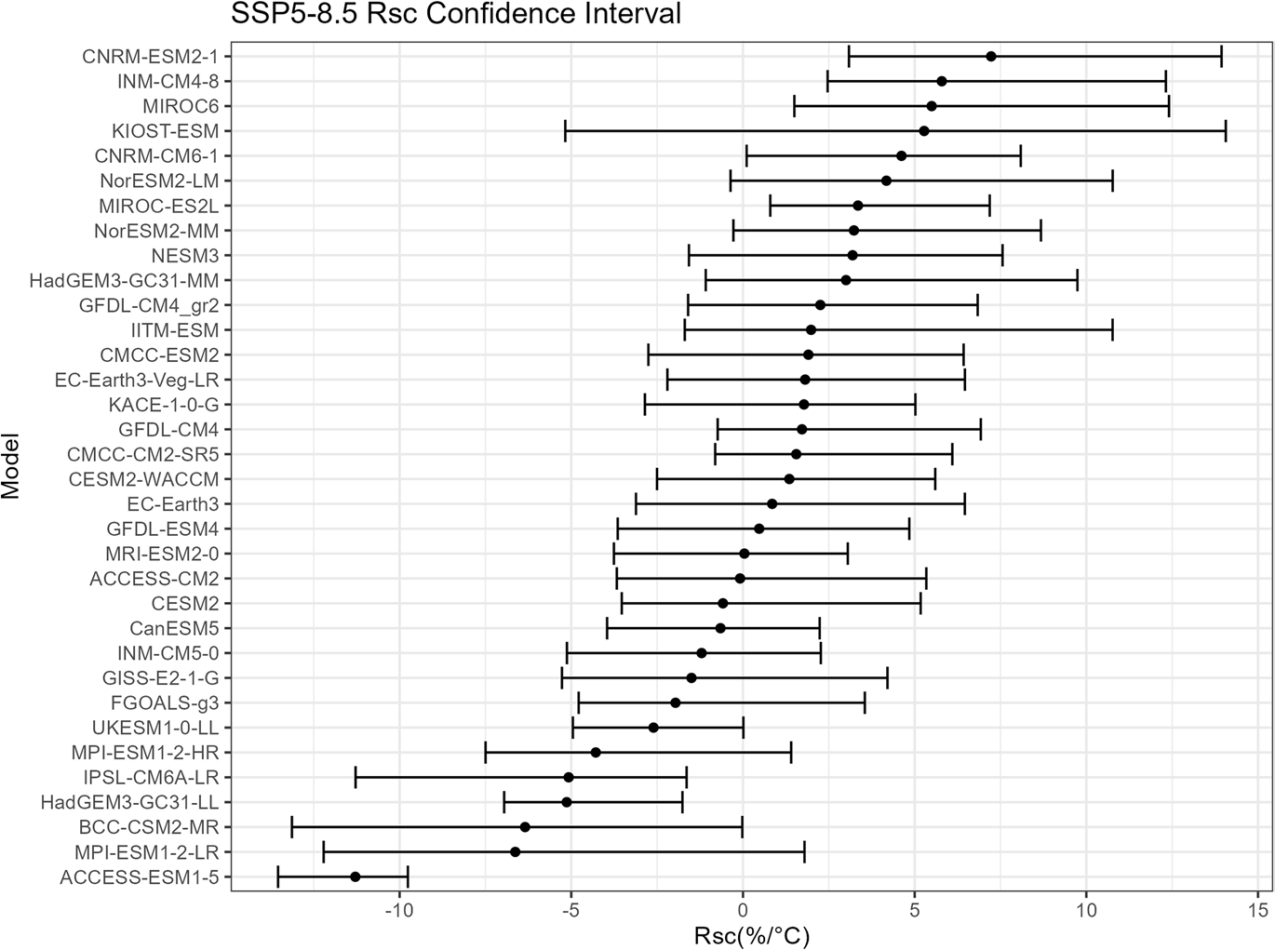
Confidence interval for ${R}_{sc}$ under SSP5-8.5

**Fig. 19:**
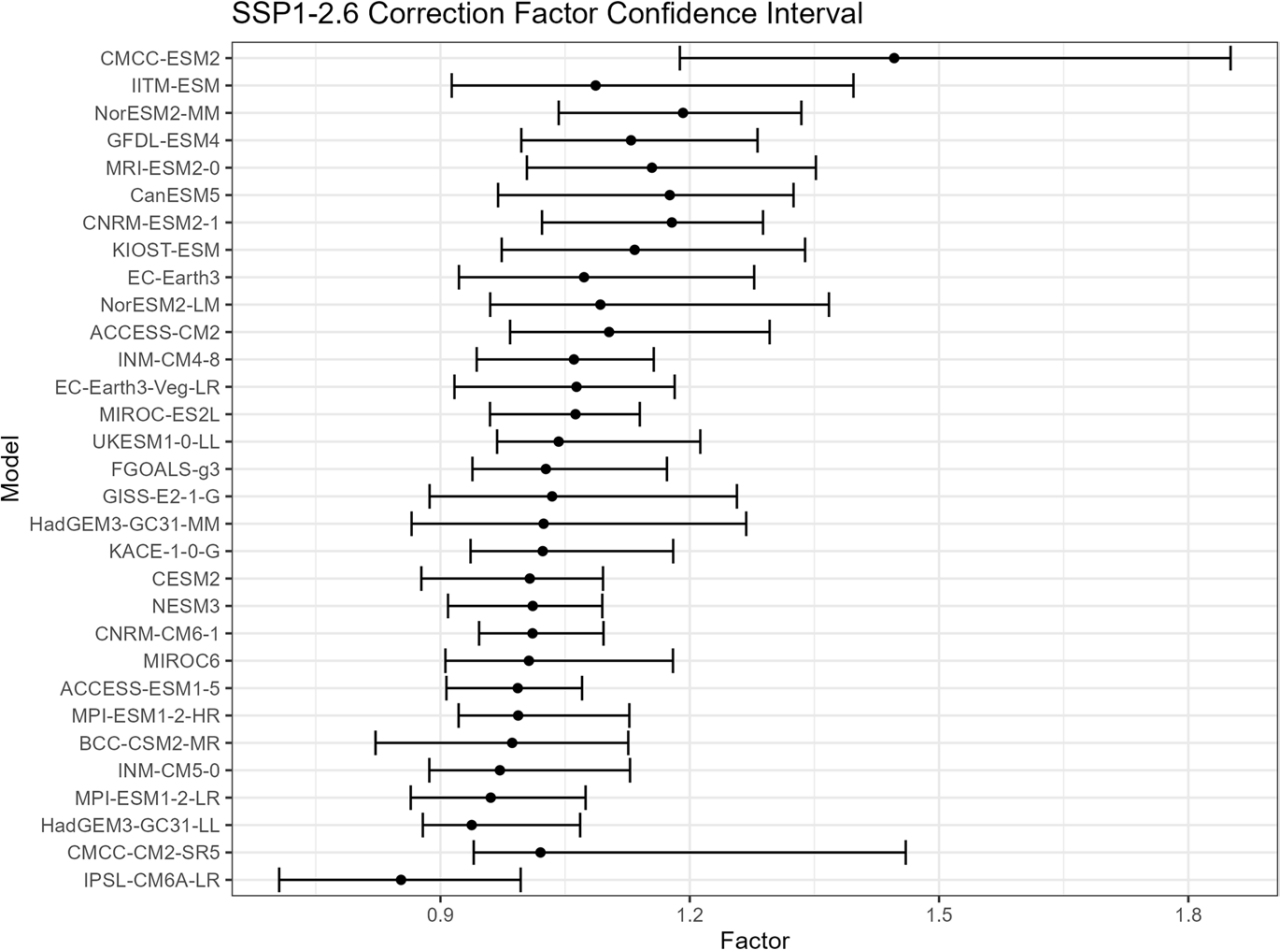
Confidence interval for correction factor under SSP1-2.6

**Fig. 20:**
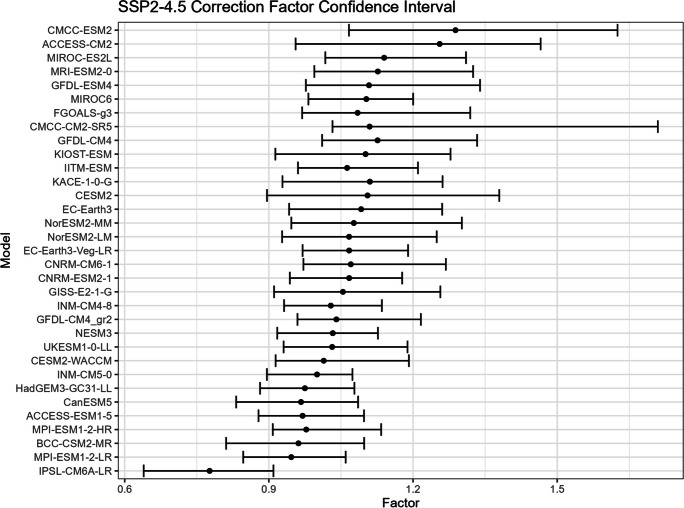
Confidence interval for correction factor under SSP2-4.5

**Fig. 21:**
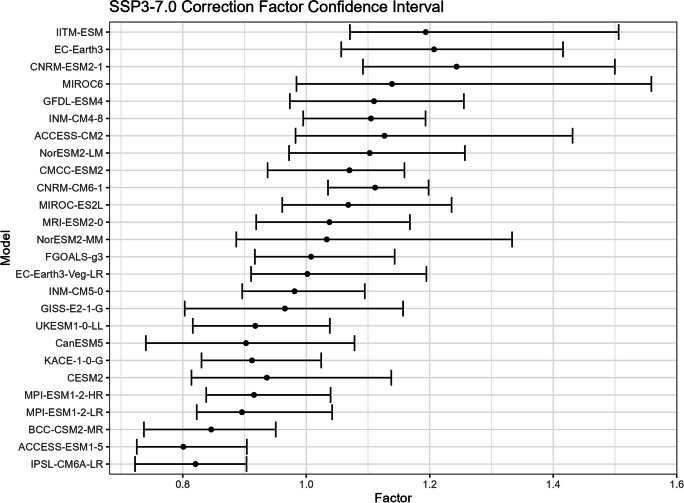
Confidence interval for correction factor under SSP3-7.0

**Fig. 22:**
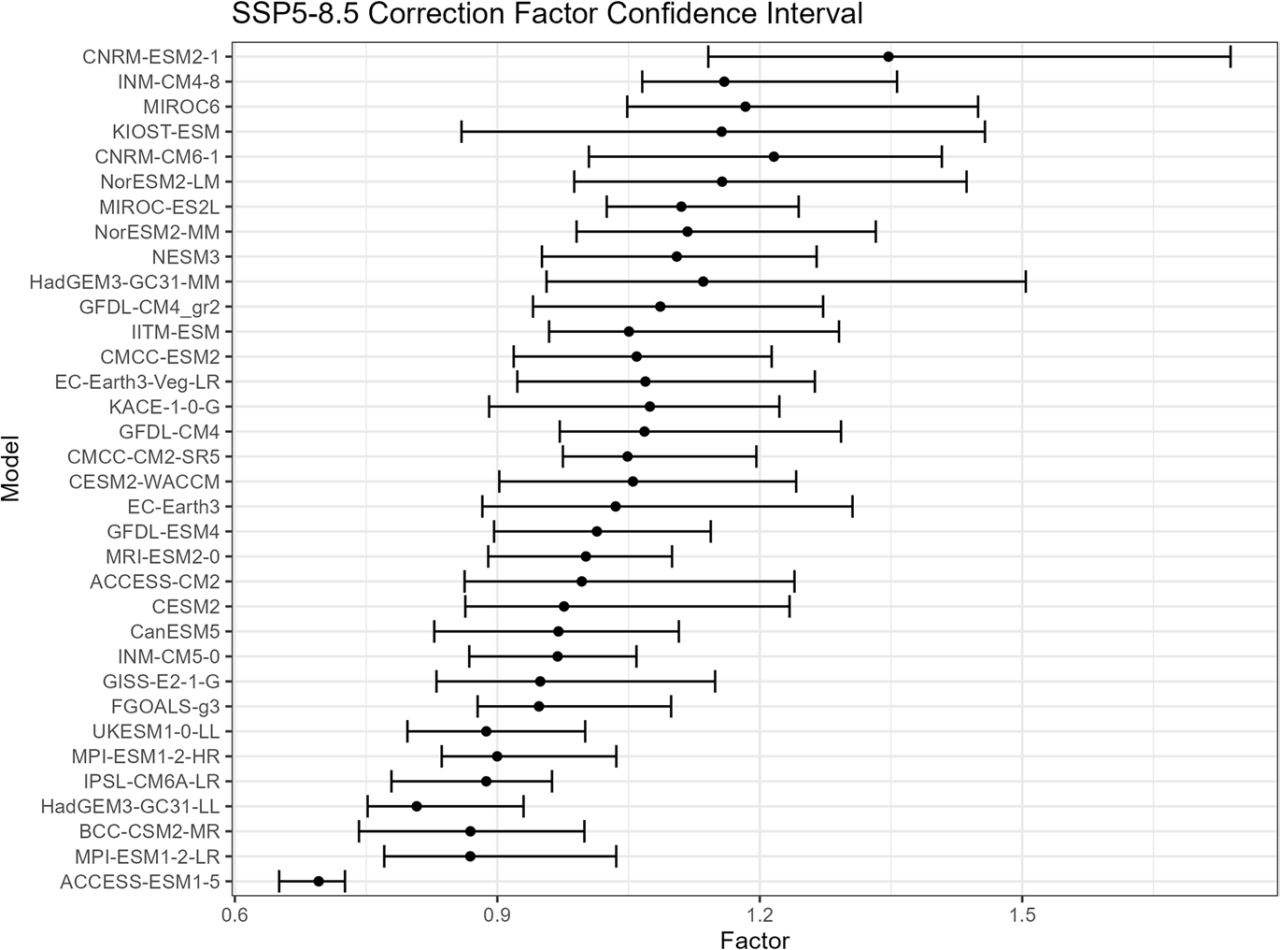
Confidence interval for correction factor under SSP5-8.5

The bootstrap method is employed in this study to estimate the confidence intervals for ${R}_{sc}$ (Figs. 15, 16, 17, and 18) and the corresponding correction factor (Figs. 19, 20, 21, and 22). The bootstrap method involves randomly sampling years from the two time periods to generate multiple realizations. Specifically, the years from 1981 to 2010 are indexed from 1 to 30, as are the years from 2071 to 2100. During each realization, indices are sampled with replacement from these ranges. Subsequently, the sampled data is composed of records corresponding to the selected indices.

For each realization, the ${R}_{sc}$ value is calculated based on the sampled data. This process is repeated 3000 times to create a distribution of ${R}_{sc}$ values. From this distribution, the 2.5% quantile and 97.5% quantile are selected to construct the 95% confidence interval.

It is noted that there are missing values (NaN) in Model TaiESM1 and CMCC-CM2-SR5 due to a specific issue encountered during the bootstrap realization process. In some instances, ${R}_{sc}$ values for these models become extremely small, reaching below − 100%, resulting in complex values for the corresponding correction factors. This occurs when the temperature difference reaches some specific values. For example, if in a particular bootstrap realization, the ${R}_{sc}$ value for Model TaiESM1 is − 200% and the temperature difference is 0.5°, the corresponding factor calculation would yield a complex value of $\sqrt{-1}=i$.

Due to these complex values caused by large standard errors, Model TaiESM1 under all SSPs and CMCC-CM2-SR5 under SSP3-7.0 are excluded from the results and analysis. By excluding these models, the analysis can focus on the remaining models and ensure the validity of the statistical estimates and interpretations.

## Discussion

Before analyzing the differences between our calculated value of ${R}_{sc}$ and the Clausius-Clapeyron relation, an intriguing finding in Table 1 deserves attention. It reveals that the value of ${R}_{sc}$ is greater in SSP1-2.6 and SSP2-4.5 compared to SSP3-7.0 and SSP5-8.5. These later SSP scenarios indicate pathways characterized by fossil-fueled development and higher greenhouse gas emissions, whereas the former SSP scenarios represent pathways with a stronger focus on sustainability.

This observation might lead us to speculate about a causal relationship between higher sustainability and a lower rainfall scaling factor. However, the influence of climate change under each SSP scenario on precipitation might not be substantial, while higher sustainability could potentially correlate with a smaller increase in temperature change. It is observed that the differences in ${R}_{sc}$ values arise primarily from differences in the denominators (temperature change) rather than the numerators (precipitation increase rate), which remain relatively similar across scenarios. To fully comprehend the underlying dynamics, it is essential to conduct rigorous causal analyses, considering other potential factors, feedback loops, and the complex interactions within the climate system.

The results of our research suggest that the confidence interval of ${R}_{sc}$ does not include the previously assumed value of 7% derived from the Clausius-Clapeyron relation. This discrepancy may be attributed to the fact that the CC relation is based on ideal environmental conditions, which may not fully apply to our specific case. Although the widely accepted 7% assumption carries weight, it is essential to acknowledge that the scaling rates between extreme precipitation and temperature are notably influenced by regional specifics, temperature dynamics, and moisture availability (Prein et al., 2016). Given the characteristics of our study site, which is a small catchment area located in an urban coastal city within a tropical region, adopting a sub-CC scaling approach is suggested. This alignment with other findings is particularly evident in conclusions where scaled rates below the traditional CC relation were identified, especially as temperatures exceeded around 20 °C. This phenomenon can potentially be attributed to the notable decrease in relative humidity experienced at higher temperatures, a correlation that has been substantiated by previous research (Drobinski et al., 2018).

Tropical areas exhibit distinct climate characteristics, with cool dry seasons, hot dry seasons, and monsoon seasons, as opposed to the traditional four-season pattern. Precipitation in these regions is heavily influenced by the monsoon season, which occurs during the summer. Our findings indicate that while climate change may have an impact on temperature, the effect on precipitation is more limited. To support this statement, we can examine the plots of temperature (Figs. 6, 7, 8, 9, and 10), which show an increasing trend. However, when we observe the plots of precipitation (Figs. 1, 2, 3, 4, and 5), we do not observe a clear upward trend. Based on this trend, we can conclude that the increase in precipitation is not significantly influenced by the increase in temperature, thus providing further evidence that ${R}_{sc}$ is not likely to be 7%.

**Fig. 23:**
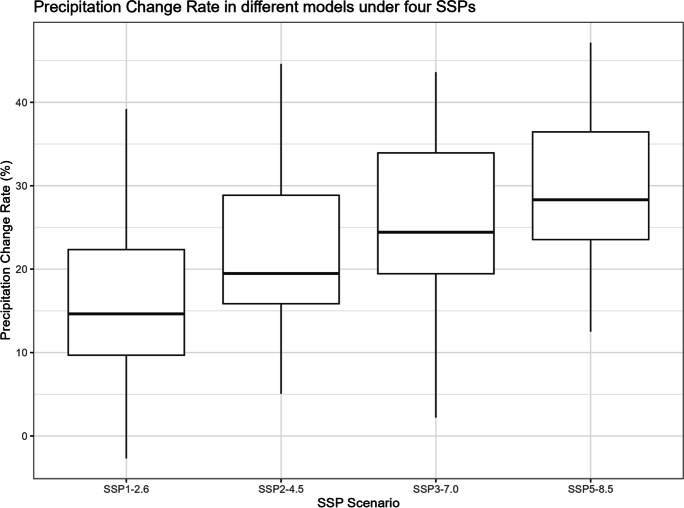
Precipitation change rate in Champaign, IL

**Fig. 24:**
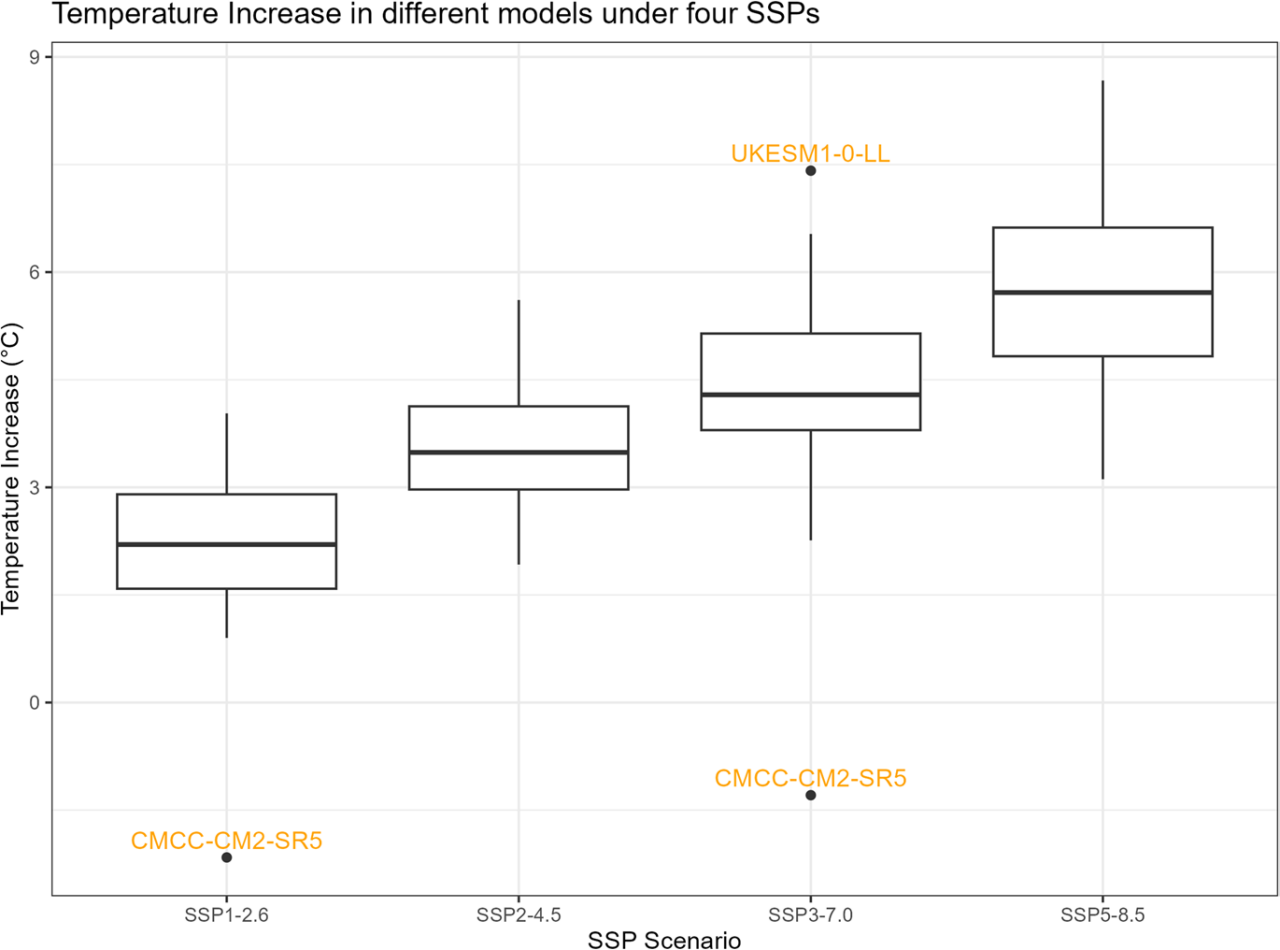
Temperature increase in Champaign, IL

**Fig. 25:**
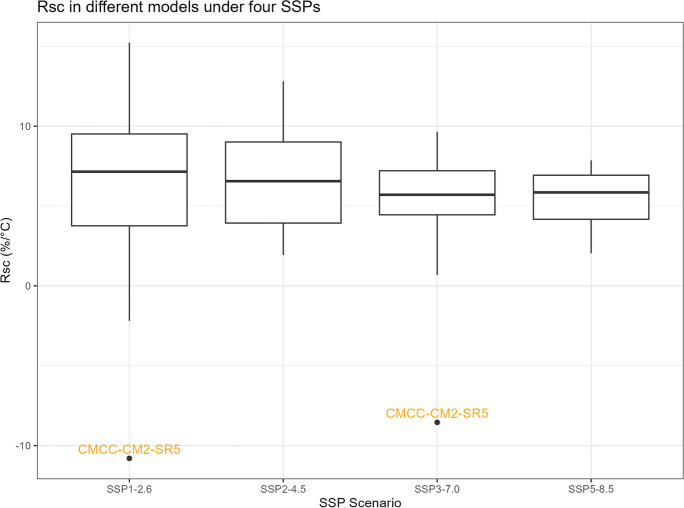
${R}_{sc}$ in Champaign, IL

**Fig. 26:**
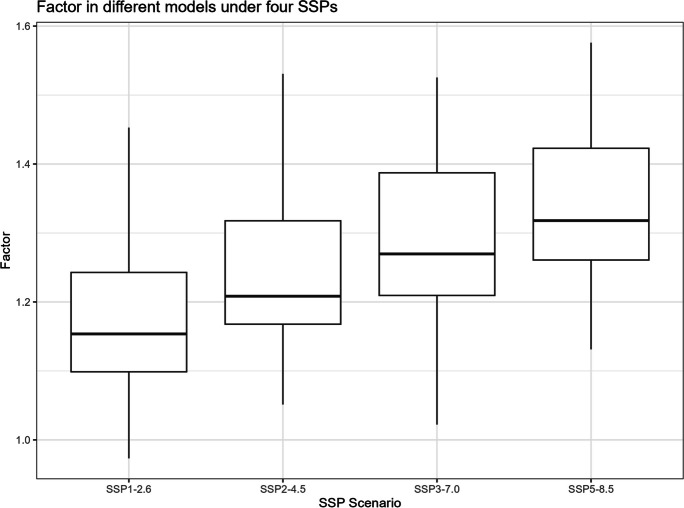
Correction factor in Champaign, IL

These findings underscore the importance of considering local climate characteristics and regional variations when interpreting the relationship between temperature and precipitation. To highlight this point, a comparative study was conducted in Champaign, Illinois, presenting a juxtaposition of a coastal city in a tropical area (Barranquilla) and an inland city in the north temperate zone (Champaign). Figures 23, 24, 25, and 26 display boxplots that illustrate variations in precipitation change rates, temperature increases, ${R}_{sc}$ values, and correction factors between these two distinct locations.

The Champaign data reveals a distinct pattern, where both the precipitation change rate and temperature increase exhibit clear upward trends as the SSP scenario transitions towards less sustainability. This has led to a relatively stable rainfall scaling factor, hovering around 7%—in alignment with the expected Clausius-Clapeyron rate. Correspondingly, the correction factor demonstrates an increasing trend as anticipated. Notably, the temperature changes observed in Champaign validate the data presented in the IPCC WGI Interactive Atlas, thereby substantiating the presence of uncertainties in the Atlas model, particularly in tropical regions.

However, it is essential to acknowledge that the contrasting results between the two locations are chiefly attributable to the divergent trends characterizing the four SSP scenarios. Presently, we attribute this divergence to the distinctive climate characteristics inherent to each region and the potential limitations within climate models. Indeed, the implications of these findings extend to the economic aspects of stormwater resources management. In regions where a sub-CC relationship is observed, the potentially lower increases in precipitation with rising temperatures could allow for the development of more cost-efficient infrastructure. This could result in significant savings in city budgets, freeing up resources for other critical areas. For example, the design of stormwater management systems could be optimized to handle the projected precipitation levels, rather than over-designing for higher increases.

While our study provides insights into the relationship between temperature and precipitation under different climate scenarios, it is important to clarify that the objective of our research is not to determine the optimal climate model. Climate models, by their nature, are simplifications of the complex climate system. Therefore, rather than focusing on a single optimal model, it may be more beneficial to consider the results from multiple models to capture a range of possible outcomes. In our case, following the methodology used in the application described in Martel et al. (2021), we used the multi-model ensemble approach. This ensemble approach can provide a more robust basis for infrastructure planning and improve the reliability of projections.

## Conclusion

Our research highlights the need to consider local climate characteristics and regional variations when evaluating the relationship between temperature and precipitation. The findings suggest that the assumption of a 7% scaling factor based on the Clausius-Clapeyron relation may not be applicable for our study location, a small urban catchment in a tropical region. Engineering and infrastructure planning guided solely by global climate assumptions could lead to substantial financial investments exceeding actual requirements. To mitigate such inefficiencies, it is paramount to conduct localized investigations and establish location-specific correction factors for precise infrastructure development.

However, our study has limitations. The primary limitation is that our research focused on two specific geographical locations, which may limit the generalizability of our findings. Additionally, our study relied on projections of climate models, which may not fully capture future climate variations due to unforeseen factors. Looking forward, we suggest expanding this research to include more diverse geographical locations. This would provide a more comprehensive understanding of the relationship between temperature and precipitation under different climate conditions.

### Electronic supplementary material

Below is the link to the electronic supplementary material.

Supplementary file1 (DOCX 41.8 KB)

## Data Availability

The datasets generated and analyzed during the current study are available from the corresponding author on reasonable request.

## Appendix

### A. Clausius-Clapeyron Relation and the 7% increase

To derive the Clausius-Clapeyron relation, we start by resembling the thermodynamic identity $dU=TdS-PdV+\mu dN$, but written for $G$ instead of $U$. By definition, $G\equiv U-TS+PV$, where $G$ is Gibbs Free energy, $T$ is absolute temperature, $S$ is the entropy, $P$ is the pressure, $V$ is the volume, and $\mu$ is the chemical potential. Now, we can express the infinitesimal change in $G$ as follows:

A1 $$dG=dU-TdS-SdT+PdV+VdP=\left(TdS-PdV+\mu dN\right)-TdS-SdT+PdV+VdPdG=-SdT+VdP+\mu dN$$

Consider a fixed amount of water at the phase boundary between liquid and vapor, where both phases are equally stable. According to the Gibbs Free energy relationship (Schroeder, 2021), the Gibbs Free energy of the liquid phase must be equal to the Gibbs Free energy of the gas phase:

$${G}_{l}={G}_{g}$$

$$d{G}_{l}=d{G}_{g}$$

From Eq. (A1), we obtain:

$$-{S}_{l}dT+{V}_{l}dP+\mu dN=-{S}_{g}dT+{V}_{g}dP+\mu dN\left({S}_{g}-{S}_{l}\right)dT=\left({V}_{g}-{V}_{l}\right)dP$$

A2 $$\frac{dP}{dT}=\frac{{S}_{g}-{S}_{l}}{{V}_{g}-{V}_{l}}=\frac{L/T}{{V}_{g}-{V}_{l}}=\frac{L}{T\Delta V}$$

Here, *L* denotes the total latent heat required to convert water from the liquid to the gas phase. Equation (A2) is known as the Clausius-Clapeyron relation.

Let $P={e}_{s}$ be the water saturation vapor pressure. By solving the previous equation, we can obtain a well-known analytical form for ${e}_{s}$.

$$\frac{d{e}_{s}}{dT}=\frac{L}{T\left({V}_{g}-{V}_{l}\right)}\approx \frac{L}{T{V}_{g}}$$

The second equality is because the volume of liquid is negligible when compared to the volume of gas, ${V}_{g}\gg {V}_{l}$. By applying the ideal gas law, $PV=nRT$, we can express ${V}_{g}$ as:

$${V}_{g}=\frac{nRT}{P}=\frac{nRT}{{e}_{s}}$$

Combining this expression with the previous equation, we have

A3 $$\frac{d{e}_{s}}{{e}_{s}}=\frac{\frac{L}{n}\bullet {e}_{s}}{R{T}^{2}}=\frac{{L}_{v}\left(T\right){e}_{s}}{{R}_{v}{T}^{2}}$$

where ${L}_{v}$ represents the specific latent heat of evaporation of water as a function of temperature, and ${R}_{v}$ represents the gas constant of water vapor $461.5J/(kg\bullet K)$. The value of ${L}_{v}$ varies with temperature: when $T=273.15K$, $L=2.501\times {10}^{6}J /kg$; when $T=373.15K$, $L=2.257\times {10}^{6}J/kg$. Assuming ${L}_{v}$ is a constant allows us to solve Eq. (A3).

$${e}_{s}={C}_{1}{\text{exp}}\left(-\frac{{L}_{v}}{{R}_{v}T}\right)$$

where ${C}_{1}$ is a constant equal to $2.53\times {10}^{11}Pa$ when $T=273.15K$ (Lawrence, 2005).

Indeed, in this application, it is important to consider the temperature dependence of both the latent heat ${L}_{v}$ and the saturation vapor pressure ${e}_{s}$. One empirical expression for ${e}_{s}$ is given by Gibbins (1990), which is known as Magnus formula:

A4 $$e_s=C_2\;\exp\left(\frac{At}{B+t}\right)$$

Initially, the parameters for this formula were determined to be relatively inaccurate. However, Alduchov and Eskridge (1996) improved the accuracy by utilizing contemporary vapor pressure measurements and suggested specific values for the coefficients. These values are as follows:

$$A=17.625, B=243.04^\circ{\rm C} , {C}_{2}=610.94Pa$$

These parameter values provide accurate results for ${e}_{s}$, with a relative error of less than 0.4% over the temperature range of − 40 to 50 °C (Lawrence, 2005).

Therefore, according to Eq. (A4), for a 1 °C increase in temperature, the scaling factor of ${e}_{s}$ can be determined as:

$$\frac{{e}_{s}\left(t+1\right)}{{e}_{s}}=\frac{{C}_{2}\;{\mathrm{exp}}\left(\frac{A\left(t+1\right)}{B+t+1}\right)}{{C}_{2}\;{\mathrm{exp}}\left(\frac{At}{B+t}\right)}={\text{exp}}\left(\frac{A\left(t+1\right)}{B+t+1}-\frac{At}{B+t}\right)\approx {\text{exp}}\left(\frac{A}{B+t}\right)$$

By evaluating this expression, we find that when *t* = 0 °C, the scaling factor is approximately 1.07527, resulting in an increase rate of 7.5%. Similarly, for *t* = 25 °C, the scaling factor is approximately 1.06797, corresponding to an increase rate of 6.8%. Based on these calculations, we can conclude that “the water-holding capacity of the atmosphere increases by approximately 7% for every 1 °C rise in temperature.” It is this conclusion that serves as the basis for recommending a value of 7% for the rainfall scaling factor (${R}_{sc}$) in Eq. (1).

### B. The summary tables of precipitation change rate and corrector factor

**Table 3:** Summary of precipitation change rate

SSPS	Min	Median	Mean	Max
SSP1-2.6	− 0.15	0.03	0.06	0.40
SSP2-4.5	− 0.23	0.07	0.06	0.27
SSP3-7.0	− 0.22	0.02	0.01	0.22
SSP5-8.5	− 0.34	0.05	0.02	0.31

Table 3

**Table 4:** Summary of correction factor

SSPS	Min	Median	Mean	Max
SSP1-2.6	0.85	1.03	1.06	1.45
SSP2-4.5	0.78	1.07	1.06	1.29
SSP3-7.0	0.80	1.02	1.02	1.24
SSP5-8.5	0.70	1.05	1.03	1.35

Table 4

## Declarations

All authors have read, understood, and have complied as applicable with the statement on “Ethical responsibilities of Authors” as found in the Instructions for Authors.

## References

[CR1] Alduchov OA, Eskridge RE (1996). Improved Magnus form approximation of saturation vapor pressure. Journal of Applied Meteorology.

[CR2] Bibi TS, Tekesa NW (2023). Impacts of climate change on IDF curves for urban stormwater management systems design: The case of Dodola Town Ethiopia. Environmental Monitoring and Assessment.

[CR3] Chen C, Zhang Q, Kashani MH, Jun C, Bateni SM, Band SS, Dash SS, Chau KW (2022). Forecast of rainfall distribution based on fixed sliding window long short-term memory. Engineering Applications of Computational Fluid Mechanics.

[CR4] Cook LM, McGinnis S, Samaras C (2020). The effect of modeling choices on updating intensity-duration-frequency curves and stormwater infrastructure designs for climate change. Climatic Change.

[CR5] CSA (Canadian Standards Association) (2019). Development, interpretation, and use of rainfall intensity-duration-frequency (IDF) information: Guideline for Canadian water resources practitioners.

[CR6] Drobinski P (2018). Scaling precipitation extremes with temperature in the Mediterranean: Past climate assessment and projection in anthropogenic scenarios. Climate Dynamics.

[CR7] Gibbins CJ (1990). A survey and comparison of relationships for the determination of the saturation vapor pressure over plane surfaces of pure water and of pure ice. Annals of GeophysIcs.

[CR8] Gregersen IB, Sunyer Pinya MA, Madsen H, Funder S, Luchner J, Rosbjerg D, Arnbjerg-Nielsen K (2014). Past, present, and future variations of extreme precipitation in Denmark: Technical report.

[CR9] IPCC (2007). Fourth Assessment report: Climate change 2007: The AR4 synthesis report.

[CR10] Jiang, C., Kang, Y., Qu, K., Long, Y., Ma, Y., & Yan, S. (2023). Towards a high-resolution modelling scheme for local-scale urban flood risk assessment based on digital aerial photogrammetry. *Engineering Applications of Computational Fluid Mechanics, 17*(1). 10.1080/19942060.2023.2240392

[CR11] Kourtis IM, Nalbantis I, Tsakiris G, Psiloglou BE, Tsihrintzis VA (2022). Updating IDF curves under climate change: Impact on rainfall-induce runoff in urban basins. Water Resources Management.

[CR12] Lawrence MG (2005). The relationship between relative humidity and the dewpoint temperature in moist air: A simple conversion and applications. Bulletin of the American Meteorological Society.

[CR13] Madsen H, Lawrence D, Lang M, Martinkova M, Kjeldsen T (2014). Review of trend analysis and climate change projections of extreme precipitation and floods in Europe. Journal of Hydrology.

[CR14] Martel, J. L., Brissette, F. P., Lucas-Picher, P., Troin, M., & Arsenault, R. (2021). Climate change and rainfall intensity–duration–frequency curves: Overview of Science and guidelines for adaptation. *Journal of Hydrologic Engineering*, *26* (10). 10.1061/(asce)he.1943-5584.0002122

[CR15] Miller RL, Schmidt GA, Nazarenko LS, Bauer SE, Kelley M, Ruedy R (2021). CMIP6 historical simulations (1850–2014) with GISS-E2.1. Journal of Advances in Modeling Earth Systems.

[CR16] NASA. (2022). *NASA Earth Exchange Global Daily Downscaled Projections (NEX-GDDP-CMIP6)*. NASA. Retrieved April-10–2023 from https://www.nccs.nasa.gov/services/data-collections/land-based-products/nex-gddp-cmip6.

[CR17] O’Gorman PA, Muller CJ (2010). How closely do changes in surface and column water vapor follow Clausius-Clapeyron scaling in climate change simulations?. Environmental Research Letters.

[CR18] Prein AF, Rasmussen RM, Ikeda K, Liu C, Clark MP, Holland GJ (2016). The future intensification of hourly precipitation extremes. Nature Climate Change.

[CR19] Schroeder DV (2021). An introduction to thermal physics.

[CR20] Şen, O., & Kahya, E. (2021). Impacts of climate change on intensity–duration–frequency curves in the rainiest city (Rize) of Turkey. *Theoretical and Applied Climatology,**144*, 1017–1030. 10.1007/s00704-021-03592-2

[CR21] Sherwood SC, Ingram W, Tsushima Y, Satoh M, Roberts M, Vidale PL, O’Gorman PA (2010). Relative humidity changes in a warmer climate. Journal of Geophysical Research: Atmospheres.

[CR22] Simmons AJ, Willett KM, Jones PD, Thorne PW, Dee DP (2010). Low-frequency variations in surface atmospheric humidity, temperature, and precipitation: Inferences from reanalyses and monthly gridded observational data sets. Journal of Geophysical Research - Atmospheres.

[CR23] Tansar H (2023). Unit-scale- and catchment-scale-based sensitivity analysis of bioretention cell for urban stormwater system management. Journal of Hydroinformatics.

[CR24] Thrasher B, Wang W, Michaelis A, Melton F, Lee T, Nemani R (2022). NASA Global Daily Downscaled Projections, CMIP6. Scientific Data.

[CR25] Westra S, Alexander LV, Zwiers FW (2013). Global increasing trends in annual maximum daily precipitation. Journal of Climate.

[CR26] Willett KM, Gillett NP, Jones PD, Thorne PW (2007). Attribution of observed surface humidity changes to human influence. Nature.

