# Supplementary material for: Climate change impacts on rainfall intensity–duration–frequency curves in local scale catchments
Source: Environ Monit Assess. 2024 Mar 15;196(4):372. doi: 10.1007/s10661-024-12532-2 (PMC10943172; doi:10.1007/s10661-024-12532-2)
Supplement: Supplementary file 1 — Supplementary file1 (DOCX 41.8 KB) [file 10661_2024_12532_MOESM1_ESM.docx]

*Supplementary Material: Detailed Bootstrap Results**

| Model | Rsc(%/°C) | SE(%/°C) | LowerCI(%/°C) | UpperCI(%/°C) | LowerfactorCI | UpperfactorCI | Pr_increase_rate | Tas_diff | Factor | SSP Scenario |
| --- | --- | --- | --- | --- | --- | --- | --- | --- | --- | --- |
| ACCESS-CM2 | -0.08459 | 2.239235 | -3.67455 | 5.339582 | 0.862576 | 1.239708 | -0.00345 | 4.076976 | 0.996556 | SSP585 |
| ACCESS-CM2 | 2.349004 | 1.868878 | -0.34916 | 7.153625 | 0.982656 | 1.431315 | 0.12063 | 5.135388 | 1.126635 | SSP370 |
| ACCESS-CM2 | 2.817633 | 2.093089 | -0.48151 | 7.594757 | 0.98379 | 1.296155 | 0.09937 | 3.526711 | 1.102958 | SSP126 |
| ACCESS-CM2 | 8.713354 | 4.145778 | -1.68228 | 14.84996 | 0.955678 | 1.465526 | 0.237247 | 2.7228 | 1.255426 | SSP245 |
| ACCESS-ESM1-5 | -11.2891 | 0.953864 | -13.5393 | -9.75884 | 0.650641 | 0.725899 | -0.34175 | 3.02722 | 0.695848 | SSP585 |
| ACCESS-ESM1-5 | -4.90454 | 1.34606 | -7.08877 | -2.25594 | 0.725502 | 0.903674 | -0.21651 | 4.41447 | 0.800916 | SSP370 |
| ACCESS-ESM1-5 | -1.38467 | 2.363043 | -6.04596 | 4.389167 | 0.878446 | 1.097989 | -0.03008 | 2.172545 | 0.970161 | SSP245 |
| ACCESS-ESM1-5 | -0.24355 | 1.400926 | -3.34196 | 2.389117 | 0.907156 | 1.070425 | -0.00698 | 2.866537 | 0.993034 | SSP126 |
| BCC-CSM2-MR | -6.345 | 3.534308 | -13.1354 | -0.02035 | 0.741902 | 0.999568 | -0.13564 | 2.137707 | 0.869244 | SSP585 |
| BCC-CSM2-MR | -4.34421 | 1.772277 | -7.81666 | -1.35589 | 0.737074 | 0.950559 | -0.16345 | 3.762567 | 0.846106 | SSP370 |
| BCC-CSM2-MR | -2.9808 | 6.393229 | -15.3242 | 7.808342 | 0.811072 | 1.098277 | -0.03854 | 1.293025 | 0.961627 | SSP245 |
| BCC-CSM2-MR | -0.60102 | 3.751887 | -8.15668 | 5.415452 | 0.821885 | 1.126043 | -0.01373 | 2.28485 | 0.986321 | SSP126 |
| CanESM5 | -1.79126 | 1.603849 | -5.16434 | 1.343136 | 0.740188 | 1.078146 | -0.10183 | 5.684682 | 0.902352 | SSP370 |
| CanESM5 | -1.23674 | 2.373205 | -6.59917 | 3.103558 | 0.83202 | 1.085685 | -0.0334 | 2.700257 | 0.966955 | SSP245 |
| CanESM5 | -0.65585 | 1.525313 | -3.95735 | 2.233586 | 0.827905 | 1.107451 | -0.0305 | 4.650384 | 0.969864 | SSP585 |
| CanESM5 | 5.047781 | 2.633017 | -0.95597 | 8.879913 | 0.969351 | 1.324917 | 0.166126 | 3.291074 | 1.175942 | SSP126 |
| CESM2 | -2.12026 | 2.838709 | -6.67482 | 4.178195 | 0.814095 | 1.137751 | -0.0653 | 3.079582 | 0.936134 | SSP370 |
| CESM2 | -0.58411 | 2.330496 | -3.52983 | 5.175255 | 0.863402 | 1.234052 | -0.02394 | 4.097797 | 0.97628 | SSP585 |
| CESM2 | 0.454466 | 3.402178 | -7.80846 | 5.688985 | 0.877001 | 1.095636 | 0.007517 | 1.653988 | 1.007528 | SSP126 |
| CESM2 | 4.029362 | 4.435646 | -4.24203 | 13.50857 | 0.896109 | 1.379457 | 0.102187 | 2.536071 | 1.105373 | SSP245 |
| CESM2-WACCM | 0.588509 | 2.740648 | -3.70973 | 7.637654 | 0.914048 | 1.191404 | 0.014127 | 2.400464 | 1.014185 | SSP245 |
| CESM2-WACCM | 1.350223 | 2.082427 | -2.50356 | 5.598759 | 0.902243 | 1.241633 | 0.053914 | 3.992951 | 1.055013 | SSP585 |
| CMCC-CM2-SR5 | -2.70048 | 16.05925 | -47.7654 | 8.826967 | 0.940014 | 1.459916 | 0.019956 | -0.739 | 1.020437 | SSP126 |
| CMCC-CM2-SR5 | 1.552862 | 1.804973 | -0.81056 | 6.096724 | 0.974978 | 1.196103 | 0.04806 | 3.094919 | 1.048846 | SSP585 |
| CMCC-CM2-SR5 | 5.802347 | 8.425101 | 1.655329 | 34.36569 | 1.032443 | 1.709296 | 0.10703 | 1.844596 | 1.109645 | SSP245 |
| CMCC-CM2-SR5 | 194.3375 | 3820.616 | -697.613 | 826.4035 | 0.863887 | NA | 0.038925 | 0.020029 | 1.021858 | SSP370 |
| CMCC-ESM2 | 1.906166 | 2.290433 | -2.7556 | 6.425609 | 0.918717 | 1.213569 | 0.058139 | 3.050065 | 1.059283 | SSP585 |
| CMCC-ESM2 | 2.044782 | 1.854472 | -2.01003 | 4.626166 | 0.93733 | 1.15901 | 0.068299 | 3.340174 | 1.069948 | SSP370 |
| CMCC-ESM2 | 13.54855 | 6.304886 | 3.157083 | 27.29314 | 1.066655 | 1.625495 | 0.27017 | 1.994091 | 1.28836 | SSP245 |
| CMCC-ESM2 | 15.25573 | 5.016259 | 6.906191 | 26.59453 | 1.188071 | 1.850833 | 0.396307 | 2.597758 | 1.446053 | SSP126 |
| CNRM-CM6-1 | 0.312452 | 1.099971 | -1.57294 | 2.758331 | 0.94646 | 1.096226 | 0.010789 | 3.453155 | 1.010831 | SSP126 |
| CNRM-CM6-1 | 2.000204 | 0.724149 | 0.647259 | 3.488872 | 1.035252 | 1.198124 | 0.106745 | 5.336706 | 1.111479 | SSP370 |
| CNRM-CM6-1 | 2.862288 | 3.285966 | -1.17581 | 10.40933 | 0.971932 | 1.26836 | 0.069056 | 2.412607 | 1.070457 | SSP245 |
| CNRM-CM6-1 | 4.616025 | 2.048922 | 0.106634 | 8.08901 | 1.00469 | 1.408103 | 0.200182 | 4.336674 | 1.216161 | SSP585 |
| CNRM-ESM2-1 | 2.638086 | 2.353051 | -2.2815 | 6.702945 | 0.943702 | 1.177387 | 0.065825 | 2.495189 | 1.067129 | SSP245 |
| CNRM-ESM2-1 | 4.107252 | 1.549769 | 1.6614 | 7.678808 | 1.091867 | 1.499754 | 0.22232 | 5.412861 | 1.243432 | SSP370 |
| CNRM-ESM2-1 | 4.895078 | 1.802538 | 0.634488 | 7.720476 | 1.02225 | 1.288172 | 0.168188 | 3.435863 | 1.178451 | SSP126 |
| CNRM-ESM2-1 | 7.226253 | 3.018754 | 3.086765 | 13.93647 | 1.141151 | 1.738132 | 0.308619 | 4.270806 | 1.347132 | SSP585 |
| EC-Earth3 | 0.849938 | 2.368241 | -3.11596 | 6.460511 | 0.882892 | 1.306014 | 0.034764 | 4.090159 | 1.035223 | SSP585 |
| EC-Earth3 | 2.908286 | 3.480469 | -3.35459 | 10.23945 | 0.922258 | 1.277545 | 0.071311 | 2.451997 | 1.072823 | SSP126 |
| EC-Earth3 | 3.9306 | 3.269951 | -2.62358 | 10.41703 | 0.942168 | 1.26069 | 0.089712 | 2.282392 | 1.091981 | SSP245 |
| EC-Earth3 | 4.452425 | 1.819688 | 1.309914 | 8.481831 | 1.056692 | 1.415829 | 0.192282 | 4.318579 | 1.206983 | SSP370 |
| EC-Earth3-Veg-LR | 0.038727 | 1.497165 | -1.95063 | 3.763549 | 0.910697 | 1.194651 | 0.001835 | 4.737164 | 1.001836 | SSP370 |
| EC-Earth3-Veg-LR | 1.810961 | 2.153084 | -2.20123 | 6.462602 | 0.922857 | 1.263006 | 0.067578 | 3.731606 | 1.069267 | SSP585 |
| EC-Earth3-Veg-LR | 2.315184 | 2.62095 | -3.30019 | 6.330766 | 0.916839 | 1.181855 | 0.06252 | 2.70043 | 1.063757 | SSP126 |
| EC-Earth3-Veg-LR | 3.259176 | 2.826896 | -1.47652 | 9.088849 | 0.97021 | 1.189692 | 0.06594 | 2.023207 | 1.06704 | SSP245 |
| FGOALS-g3 | -1.96503 | 2.150936 | -4.78477 | 3.548901 | 0.877491 | 1.098668 | -0.05327 | 2.710823 | 0.947623 | SSP585 |
| FGOALS-g3 | 0.182472 | 1.306368 | -2.02125 | 3.17729 | 0.916722 | 1.143106 | 0.007869 | 4.312275 | 1.007892 | SSP370 |
| FGOALS-g3 | 1.060864 | 2.270574 | -2.50229 | 6.516872 | 0.938473 | 1.17253 | 0.026692 | 2.5161 | 1.026907 | SSP126 |
| FGOALS-g3 | 6.060975 | 6.070788 | -2.29602 | 21.72381 | 0.969322 | 1.319085 | 0.083687 | 1.380749 | 1.084641 | SSP245 |
| GFDL-CM4 | 1.718704 | 2.007706 | -0.73702 | 6.924938 | 0.971403 | 1.292916 | 0.066549 | 3.872026 | 1.068209 | SSP585 |
| GFDL-CM4 | 5.647366 | 3.686524 | 0.518759 | 14.9005 | 1.010853 | 1.333366 | 0.122156 | 2.163062 | 1.12618 | SSP245 |
| GFDL-CM4_gr2 | 1.895189 | 3.02799 | -1.93367 | 10.03115 | 0.959569 | 1.216388 | 0.040101 | 2.115945 | 1.040526 | SSP245 |
| GFDL-CM4_gr2 | 2.253492 | 2.04569 | -1.59865 | 6.834845 | 0.940803 | 1.272423 | 0.083699 | 3.714181 | 1.086292 | SSP585 |
| GFDL-ESM4 | 0.4724 | 2.050221 | -3.64779 | 4.84391 | 0.896266 | 1.143939 | 0.0138 | 2.921172 | 1.013862 | SSP585 |
| GFDL-ESM4 | 2.71716 | 1.569928 | -0.69179 | 6.049251 | 0.973407 | 1.255237 | 0.105442 | 3.880582 | 1.109639 | SSP370 |
| GFDL-ESM4 | 6.346332 | 3.261902 | -0.13365 | 13.52203 | 0.997295 | 1.281576 | 0.125454 | 1.976796 | 1.129341 | SSP126 |
| GFDL-ESM4 | 6.759255 | 5.258118 | -1.56673 | 21.04009 | 0.97702 | 1.339456 | 0.106303 | 1.572708 | 1.108342 | SSP245 |
| GISS-E2-1-G | -1.50013 | 2.369279 | -5.27098 | 4.207627 | 0.830447 | 1.148941 | -0.05185 | 3.456272 | 0.9491 | SSP585 |
| GISS-E2-1-G | -0.74555 | 1.927942 | -4.57776 | 3.109109 | 0.803139 | 1.156851 | -0.03513 | 4.712055 | 0.965352 | SSP370 |
| GISS-E2-1-G | 1.042786 | 2.797936 | -3.59851 | 7.556875 | 0.886913 | 1.256732 | 0.034006 | 3.261088 | 1.034409 | SSP126 |
| GISS-E2-1-G | 2.378868 | 3.794863 | -4.08052 | 10.79772 | 0.910891 | 1.256896 | 0.053311 | 2.241031 | 1.0541 | SSP245 |
| HadGEM3-GC31-LL | -5.13222 | 1.19383 | -6.95526 | -1.76305 | 0.751779 | 0.930024 | -0.20784 | 4.04969 | 0.807864 | SSP585 |
| HadGEM3-GC31-LL | -1.76509 | 1.436711 | -3.56016 | 1.806468 | 0.878748 | 1.068112 | -0.06385 | 3.617452 | 0.93761 | SSP126 |
| HadGEM3-GC31-LL | -0.92378 | 1.895195 | -4.48329 | 2.808385 | 0.881733 | 1.078005 | -0.0252 | 2.728379 | 0.974997 | SSP245 |
| HadGEM3-GC31-MM | 0.987496 | 3.842847 | -5.86388 | 10.02793 | 0.865254 | 1.267934 | 0.024005 | 2.430928 | 1.024175 | SSP126 |
| HadGEM3-GC31-MM | 3.001988 | 2.549578 | -1.08431 | 9.740169 | 0.956261 | 1.504067 | 0.128879 | 4.29312 | 1.135397 | SSP585 |
| IITM-ESM | 1.983906 | 3.202277 | -1.69596 | 10.76478 | 0.959173 | 1.290608 | 0.049731 | 2.506738 | 1.050477 | SSP585 |
| IITM-ESM | 4.24408 | 4.631065 | -2.70938 | 13.56236 | 0.960643 | 1.210266 | 0.062309 | 1.468136 | 1.062923 | SSP245 |
| IITM-ESM | 8.741892 | 4.766551 | 3.336644 | 21.61156 | 1.070743 | 1.505884 | 0.184531 | 2.110881 | 1.193519 | SSP370 |
| IITM-ESM | 9.658696 | 14.92728 | -9.99564 | 46.4592 | 0.913552 | 1.397044 | 0.087184 | 0.902647 | 1.086788 | SSP126 |
| INM-CM4-8 | 2.061495 | 3.613619 | -4.90727 | 9.561107 | 0.931675 | 1.135161 | 0.028783 | 1.396234 | 1.0289 | SSP245 |
| INM-CM4-8 | 2.50643 | 2.284592 | -2.40996 | 6.434213 | 0.943633 | 1.156687 | 0.059631 | 2.379136 | 1.060665 | SSP126 |
| INM-CM4-8 | 2.54866 | 1.190898 | -0.12095 | 4.554413 | 0.995159 | 1.193175 | 0.100889 | 3.958494 | 1.104756 | SSP370 |
| INM-CM4-8 | 5.78919 | 2.602423 | 2.466068 | 12.31593 | 1.065652 | 1.356903 | 0.152157 | 2.628293 | 1.159415 | SSP585 |
| INM-CM5-0 | -1.20487 | 1.839811 | -5.12625 | 2.267391 | 0.867933 | 1.05905 | -0.03137 | 2.603417 | 0.968934 | SSP585 |
| INM-CM5-0 | -1.15536 | 2.754463 | -4.6233 | 5.20784 | 0.886591 | 1.128144 | -0.02874 | 2.487169 | 0.971511 | SSP126 |
| INM-CM5-0 | -0.48522 | 1.246672 | -2.67986 | 2.379026 | 0.896309 | 1.094725 | -0.01908 | 3.931248 | 0.98106 | SSP370 |
| INM-CM5-0 | 0.0214 | 3.275787 | -6.96266 | 5.425661 | 0.895933 | 1.073913 | 0.000308 | 1.440071 | 1.000308 | SSP245 |
| IPSL-CM6A-LR | -18.715 | 7.568674 | -35.9836 | -6.75568 | 0.639516 | 0.909527 | -0.22817 | 1.219183 | 0.776759 | SSP245 |
| IPSL-CM6A-LR | -6.6474 | 3.67296 | -14.4393 | -0.14691 | 0.705915 | 0.996546 | -0.15412 | 2.318546 | 0.852583 | SSP126 |
| IPSL-CM6A-LR | -5.1287 | 1.5301 | -8.51062 | -2.69113 | 0.722776 | 0.903275 | -0.19252 | 3.753707 | 0.820675 | SSP370 |
| IPSL-CM6A-LR | -5.07533 | 2.516457 | -11.2816 | -1.64248 | 0.778987 | 0.962512 | -0.11644 | 2.294261 | 0.887364 | SSP585 |
| KACE-1-0-G | -1.81342 | 1.047171 | -3.63183 | 0.466964 | 0.83034 | 1.02428 | -0.0912 | 5.029079 | 0.912073 | SSP370 |
| KACE-1-0-G | 0.616561 | 1.547655 | -1.80461 | 4.414502 | 0.936224 | 1.180056 | 0.022911 | 3.715951 | 1.023104 | SSP126 |
| KACE-1-0-G | 1.776155 | 2.034058 | -2.85688 | 5.020362 | 0.890596 | 1.222402 | 0.072396 | 4.075986 | 1.074398 | SSP585 |
| KACE-1-0-G | 4.14205 | 2.988419 | -2.9271 | 9.26672 | 0.92833 | 1.261492 | 0.106699 | 2.576001 | 1.110209 | SSP245 |
| KIOST-ESM | 4.823266 | 3.2624 | -1.01314 | 11.40793 | 0.9737 | 1.338811 | 0.128522 | 2.664622 | 1.133736 | SSP126 |
| KIOST-ESM | 5.275882 | 5.034435 | -5.17382 | 14.06003 | 0.859056 | 1.457363 | 0.149111 | 2.826279 | 1.156399 | SSP585 |
| KIOST-ESM | 5.418358 | 4.869195 | -4.8516 | 14.35633 | 0.913523 | 1.278049 | 0.099049 | 1.828033 | 1.101265 | SSP245 |
| MIROC-ES2L | 1.512749 | 1.437507 | -0.92531 | 4.930134 | 0.961199 | 1.235373 | 0.06633 | 4.384732 | 1.068049 | SSP370 |
| MIROC-ES2L | 2.224565 | 1.601135 | -1.55347 | 4.892164 | 0.959622 | 1.139885 | 0.061353 | 2.757986 | 1.06256 | SSP126 |
| MIROC-ES2L | 3.349625 | 1.481988 | 0.794092 | 7.185392 | 1.025091 | 1.244473 | 0.10641 | 3.176785 | 1.110341 | SSP585 |
| MIROC-ES2L | 7.621858 | 3.921943 | 0.920762 | 16.59577 | 1.017491 | 1.310234 | 0.135807 | 1.781805 | 1.139831 | SSP245 |
| MIROC6 | 0.213691 | 2.17854 | -3.1232 | 5.776378 | 0.905945 | 1.179798 | 0.006454 | 3.020256 | 1.006468 | SSP126 |
| MIROC6 | 2.949806 | 2.844714 | -0.35533 | 10.4525 | 0.984185 | 1.558924 | 0.131918 | 4.472091 | 1.138839 | SSP370 |
| MIROC6 | 5.494807 | 2.663138 | 1.496901 | 12.4065 | 1.048519 | 1.449479 | 0.173165 | 3.151429 | 1.183617 | SSP585 |
| MIROC6 | 6.298952 | 3.688492 | -1.03757 | 12.94047 | 0.982294 | 1.2002 | 0.100858 | 1.601185 | 1.102752 | SSP245 |
| MPI-ESM1-2-HR | -4.28601 | 2.293173 | -7.49774 | 1.404586 | 0.836407 | 1.036029 | -0.10328 | 2.409803 | 0.899818 | SSP585 |
| MPI-ESM1-2-HR | -2.59568 | 1.633237 | -5.20876 | 1.134341 | 0.838044 | 1.039399 | -0.08736 | 3.365444 | 0.915294 | SSP370 |
| MPI-ESM1-2-HR | -1.59598 | 4.191518 | -7.06449 | 9.117336 | 0.908191 | 1.133691 | -0.02222 | 1.392244 | 0.97785 | SSP245 |
| MPI-ESM1-2-HR | -0.33682 | 2.64172 | -4.19788 | 6.468919 | 0.921848 | 1.127325 | -0.00649 | 1.92814 | 0.993516 | SSP126 |
| MPI-ESM1-2-LR | -6.63099 | 3.926676 | -12.2117 | 1.792925 | 0.770756 | 1.035929 | -0.13559 | 2.044787 | 0.869103 | SSP585 |
| MPI-ESM1-2-LR | -5.16551 | 5.317899 | -15.3559 | 5.623589 | 0.846664 | 1.059834 | -0.05342 | 1.034247 | 0.946624 | SSP245 |
| MPI-ESM1-2-LR | -2.90278 | 1.607937 | -5.27771 | 1.062845 | 0.822563 | 1.041992 | -0.10839 | 3.733888 | 0.895842 | SSP370 |
| MPI-ESM1-2-LR | -1.59818 | 2.189197 | -5.79116 | 2.926454 | 0.864222 | 1.074691 | -0.03998 | 2.501458 | 0.960501 | SSP126 |
| MRI-ESM2-0 | 0.040332 | 1.782879 | -3.75812 | 3.052721 | 0.889582 | 1.099711 | 0.001265 | 3.136847 | 1.001266 | SSP585 |
| MRI-ESM2-0 | 0.877876 | 1.51072 | -1.97099 | 3.759746 | 0.91899 | 1.167809 | 0.037023 | 4.217355 | 1.037549 | SSP370 |
| MRI-ESM2-0 | 6.059931 | 3.221017 | 0.16196 | 13.1027 | 1.004046 | 1.351872 | 0.14799 | 2.442101 | 1.154513 | SSP126 |
| MRI-ESM2-0 | 6.979472 | 4.261512 | -0.29676 | 16.59906 | 0.99466 | 1.324989 | 0.123416 | 1.768272 | 1.126707 | SSP245 |
| NESM3 | 0.348396 | 1.537131 | -3.01191 | 2.90102 | 0.90908 | 1.09477 | 0.011025 | 3.164572 | 1.011067 | SSP126 |
| NESM3 | 1.774218 | 2.881736 | -4.85482 | 6.487613 | 0.917507 | 1.126947 | 0.032822 | 1.849936 | 1.033069 | SSP245 |
| NESM3 | 3.192301 | 2.287795 | -1.5741 | 7.559699 | 0.95102 | 1.265011 | 0.101542 | 3.180845 | 1.105121 | SSP585 |
| NorESM2-LM | 2.144355 | 1.51906 | -0.59946 | 5.104339 | 0.972188 | 1.256901 | 0.098869 | 4.610685 | 1.102769 | SSP370 |
| NorESM2-LM | 2.898101 | 3.028778 | -1.34368 | 10.4021 | 0.959857 | 1.367556 | 0.089751 | 3.09689 | 1.092507 | SSP126 |
| NorESM2-LM | 3.337298 | 4.299199 | -3.75975 | 12.55388 | 0.927592 | 1.249279 | 0.065845 | 1.972989 | 1.066913 | SSP245 |
| NorESM2-LM | 4.177355 | 2.928621 | -0.36342 | 10.76739 | 0.987937 | 1.436363 | 0.148792 | 3.561864 | 1.156928 | SSP585 |
| NorESM2-MM | 0.871258 | 2.480305 | -3.16222 | 8.129496 | 0.886675 | 1.33336 | 0.032864 | 3.77198 | 1.033263 | SSP370 |
| NorESM2-MM | 3.231511 | 2.332803 | -0.27956 | 8.678314 | 0.99061 | 1.33263 | 0.112751 | 3.489112 | 1.117359 | SSP585 |
| NorESM2-MM | 3.688143 | 3.758345 | -2.65138 | 13.71238 | 0.946799 | 1.301629 | 0.075336 | 2.042656 | 1.076785 | SSP245 |
| NorESM2-MM | 7.549095 | 2.744765 | 1.792118 | 13.14856 | 1.042352 | 1.334345 | 0.182082 | 2.411972 | 1.191886 | SSP126 |
| TaiESM1 | -49.9325 | 4299.942 | -1909.8 | 1908.6 | 0.925711 | NA | 0.056483 | -0.11312 | 1.081399 | SSP585 |
| TaiESM1 | -31.8191 | 47.79788 | -109.208 | -8.84278 | 1.084793 | NA | 0.234691 | -0.73758 | 1.326437 | SSP126 |
| TaiESM1 | -14.7858 | 11.12146 | -40.2677 | 0.942412 | 0.986745 | 1.810991 | 0.188073 | -1.27199 | 1.22571 | SSP245 |
| TaiESM1 | 19.97943 | 9593.095 | -362.442 | 360.296 | 0.940755 | NA | 0.050951 | 0.255018 | 1.047547 | SSP370 |
| UKESM1-0-LL | -2.60565 | 1.200919 | -4.95334 | 0.0111 | 0.797254 | 1.000473 | -0.11796 | 4.526919 | 0.887346 | SSP585 |
| UKESM1-0-LL | -1.584 | 1.13419 | -3.73293 | 0.67829 | 0.816209 | 1.03817 | -0.0855 | 5.397854 | 0.917423 | SSP370 |
| UKESM1-0-LL | 1.133472 | 2.394181 | -2.6425 | 6.375244 | 0.930712 | 1.188505 | 0.031452 | 2.774803 | 1.031769 | SSP245 |
| UKESM1-0-LL | 1.146425 | 1.641479 | -0.88079 | 5.322406 | 0.968171 | 1.212692 | 0.041648 | 3.632828 | 1.04228 | SSP126 |

*Column Description

The first column is the name of the model.

The second column Rsc(%/°C) represents the calculated $R_{sc}$ for each model under given SSP scenario.

The third column SE(%/°C) represents the standard error of the calculated $R_{sc}$.

The fourth column LowerCI(%/°C) represents the lower bound of the estimated confidence interval for $R_{sc}$.

The fifth column UpperCI(%/°C) represents the upper bound of the estimated confidence interval for $R_{sc}$.

The sixth column LowerfactorCI represents the lower bound of the estimated confidence interval for the correction factor CF.

The seventh column UpperfactorCI represents the upper bound of the estimated confidence interval for the correction factor CF.

The eighth column Pr_increase_rate represents the rate of increase in precipitation, which is also the numerator when calculate the $R_{sc}$.

The ninth column Tas_diff represents the temperature increase, which is also the denominator when calculate the $R_{sc}$.

The tenth column Factor represents the value of the correction factor calculated from the corresponding $R_{sc}$ and temperature increase.

The eleventh column SSP Scenario represents the SSP scenario of the row.
